# Identification and validation of G protein-coupled receptors modulating flow-dependent signaling pathways in vascular endothelial cells

**DOI:** 10.3389/fmolb.2023.1198079

**Published:** 2023-06-08

**Authors:** Dike Qiu, Ke Xu, Namjin Chung, Jennifer Robbins, Robert Luo, Michael Lawrence, Aiqing He, Fei Yu, Andrew Alt, Michael M. Miller, Jon Hangeland, John N. Feder, Dietmar Seiffert, Brian J. Arey

**Affiliations:** Research and Early Development, Bristol Myers Squibb Company, Princeton, NJ, United States

**Keywords:** G protein coupled receptor, KLF2, laminar flow, endothelial cells, LGR4, GPR101, beta-arrestin, drug discovery

## Abstract

Vascular endothelial cells are exposed to mechanical forces due to their presence at the interface between the vessel wall and flowing blood. The patterns of these mechanical forces (laminar vs. turbulent) regulate endothelial cell function and play an important role in determining endothelial phenotype and ultimately cardiovascular health. One of the key transcriptional mediators of the positive effects of laminar flow patterns on endothelial cell phenotype is the zinc-finger transcription factor, krüppel-like factor 2 (KLF2). Given its importance in maintaining a healthy endothelium, we sought to identify endothelial regulators of the KLF2 transcriptional program as potential new therapeutic approaches to treating cardiovascular disease. Using an approach that utilized both bioinformatics and targeted gene knockdown, we identified endothelial GPCRs capable of modulating KLF2 expression. Genetic screening using siRNAs directed to these GPCRs identified 12 potential GPCR targets that could modulate the KLF2 program, including a subset capable of regulating flow-induced KLF2 expression in primary endothelial cells. Among these targets, we describe the ability of several GPCRs (GPR116, SSTR3, GPR101, LGR4) to affect KLF2 transcriptional activation. We also identify these targets as potential validated targets for the development of novel treatments targeting the endothelium. Finally, we highlight the initiation of drug discovery efforts for LGR4 and report the identification of the first known synthetic ligands to this receptor as a proof-of-concept for pathway-directed phenotypic screening to identify novel drug targets.

## Introduction

The endothelium lining the surface of blood vessels plays a crucial role in vascular homeostasis through regulation of vascular tone, release of inflammatory/thrombotic regulators and barrier function. Studies over the last several decades have clearly demonstrated that the maintenance of a healthy endothelium is critical for a healthy cardiovascular system (Alexander et al., 2020). Beyond its role in maintaining vascular tone, the endothelium is recognized as a key point of interaction between the cardiovascular system and other physiological systems such as metabolism and inflammation. This is borne out through the link between endothelial dysfunction and cardiovascular disease associated with a spectrum of pathological states (Alexander et al., 2020).

Due to its direct anatomical association with flowing blood, the endothelium is subjected to the force of blood flow across its surface. Indeed, one of the key regulators of endothelial function/dysfunction has been found to be the type (laminar vs. turbulent) and intensity of shear force directed at the vascular surface. High shear force (>12 dynes/cm^2^), laminar flow has been found to induce a transcriptional program that promotes an endothelial cell phenotype that has improved barrier function and relaxation properties and is both anti-inflammatory and anti-thrombotic (Traub and Berk, 1998; Nagel et al., 1999; Yamashiro and Yanagisawa, 2020). One of the main effectors of this transcriptional program is the zinc-finger transcription factor, krüppel-like factor 2 (KLF2) (SenBanerjee et al., 2004; Parmar et al., 2006).

KLF2 expression is rapidly stimulated by laminar shear force (>12 dynes/cm^2^) but reduced by turbulent shear (<5 dynes/cm^2^) (SenBanerjee et al., 2004). Elevation of KLF2 expression in endothelial cells results in a concomitant decrease in inflammatory genes encoding proteins such as MCP-1 and CXCL12 (Parmar et al., 2006). Due to its role as a key mediator regulating endothelial function, KLF2 would appear to be an attractive therapeutic target for treating atherosclerosis. However, transcription factors have historically been intractable targets for the development of safe and efficacious drugs due to their pleiotropic activities across tissues, inherent structural fluidity, and lack of hydrophobic pockets amenable to binding small molecules.

Conversely, G protein-coupled receptors (GPCRs) remain today the most druggable target class for the development of novel therapeutics. This is due to their role as key receptive mediators of endogenous and exogenous factors. GPCRs respond to many different types of signals including peptides and proteins, biogenic amines, photons and ions. Among the diversity of naturally occurring agonists for GPCRs are non-traditional activators such as cell adhesion molecules. Recently, GPCRs have also been found to be mechano-transducers capable of perceiving physical forces imposed on the surface of cells. Indeed, several reports have described the responsiveness of various GPCRs to fluid shear forces (Mederosy Schnitzler et al., 2008; White et al., 2014; Wang et al., 2018; Xu et al., 2018). Given the nexus of flow-dependent endothelial function and the KLF2 transcriptional program in endothelial cells, it would seem logical that GPCRs exist on the surface of the endothelium that can potentially regulate the KLF2 transcriptional program and therefore represent attractive and druggable targets for development of novel therapeutics to treat cardiovascular disease through enhancing and maintaining a healthy endothelial phenotype.

In this manuscript, we describe our effort to screen and identify GPCRs capable of modulating KLF2 expression within endothelial cells as a means for the identification of novel drug targets for this purpose. We highlight the validation of several of these targets and describe the initial drug discovery efforts around one such target, LGR4.

## Materials and methods

### HUVEC/HAEC/EA-hy926 cell culture

Human umbilical vein endothelial cells (HUVEC) (Lonza #CC-2517) or human aortic endothelial cells (HAEC) (Lonza #CC-2535) were cultured at 37°C and 5% CO_2_ in endothelial cell growth medium containing 2% FBS and additional growth factors (EGM-2 Bullet Kit, Lonza #CC-3162). HUVECs were split 1:4 when they obtained approximately 80% confluency. Only cells passaged less than six times were used in experiments. For 96 well plate assays, cells were seeded at 30,000 cells per well in Collagen I coated 96 well plates. Twenty-four hours after seeding, growth medium was replaced with serum-free medium for an additional 24 h. The cells were subsequently exposed to either DMSO or treatment compounds in serum-free growth medium for the indicated times. The final concentration of DMSO for controls and compound treatments was 0.1%. EA-Hy926 cells (ATCC, #CRL-2922) were maintained in DMEM (ATCC, #30-2002), 10% FBS (Sigma D4135-500), GlutMax I 1X, 0.5 μg/mL G418, 0.25 mg/mL Zeocin and seeded into 96 well plates at 15,000 cells per well. Experiments with EA.hy926 cells were conducted in DMEM without serum and supplements.

### EA-hy926 cell KLF2 promoter-luciferase reporter cell line

In order to develop a stable recombinant cell line for use in assessing activation of KLF2 expression, we transfected the endothelial hybrid cell line, EA-hy926, with a pcDNA expression plasmid containing 1,000 bp upstream of the transcriptional start site (which includes the putative flow-sensitive response element and 3′-UTR) driving the expression of the firefly luciferase gene (G418 resistant). Following selection and expansion of a stable clone, the cell line was subsequently transfected with a pcDNA expression plasmid containing the sequence for the human LGR4 (containing Zeocin resistance). A stable clone was identified under dual selection and expanded in DMEM (ATCC, #30-2002), 10% FBS (Sigma D4135-500), GlutMax I 1X, 0.5 μg/mL G418, 0.25 mg/mL Zeocin. Compounds were studied for their ability to induce luciferase expression in DMEM without any supplements. Briefly, cells were seeded into 96 well plates at 15,000 cells/well and grown in 200 μL of complete growth medium for 48 h. The cells were washed three times with 200 μL of DMEM without supplements then incubated in 200 μL of assay medium containing compounds at increasing concentrations or DMSO. After 16 h of incubation, the cells were lysed in 60 μL of 1X lysis reagent (Promega #E153A), and luciferase reaction performed using the luciferase assay system from Promega (#E1504). The luciferase signal was measured on an EnVision Multimode Plate reader (Perkin Elmer) and data analyzed.

### Gene expression

To measure expression of endogenous KLF2 expression in HUVECs or HAECs we employed a qRT-PCR assay. Total RNA from cells challenged from 1–24 h in 96 well plates were isolated using the ABI 6100 Nucleic Acid Prep Station according to the manufacturer’s instructions. A total of 5–10 ng RNA was reverse transcribed in a volume of 20 μL using qScript cDNA synthesis kit (Quanta Bio #95047-500). Real-time PCR analyses were carried out with SYBR Green (Quanta Bio #95055-024). Human L30 mRNA was used as the internal standard. The RT-PCR and the data collection were performed on 7900HT Sequence detection systems (Applied Biosystems). Relative expression of KLF2 was calculated and normalized relative to human L30. The primer sequences used were:

Human KLF2: Forward Primer: CTA​CAC​CAA​GAG​TTC​GCA​TCT​G, Reverse Primer: CCG​TGT​GCT​TTC​GGT​AGT​G

Human L30:

Forward Primer: GCT​GGA​GTC​GAT​CAA​CTC​TAG​G, Reverse Primer: CCA​ATT​TCG​CTT​TGC​CTT​GTC

Human LGR4:

Forward Primer: GGC​AAC​GAC​CTT​TCT​TTT​ATC​CA, Reverse Primer: CAC​TGG​GTA​CTG​TTT​TCA​ACT​GA

Human SSTR3:

Forward Primer: CTG​GGT​AAC​TCG​CTG​GTC​AT, Reverse Primer: CAG​GCA​GAA​TAT​GCT​GGT​GA

Human GPR116:

Forward Primer: CGA​GCC​GTT​GCC​ACA​AAA​AG, Reverse Primer: ATG​TCG​GTA​ATT​TGG​TCA​GTG​TT

Human GPR101:

Forward Primer: AGA​CGA​CAT​CAA​TTT​CAG​TGA​GG, Reverse Primer: GCT​GTT​GCT​GTT​ACG​ACG​ACT

In some instances, quantitative gene expression profiling on a subset of known endothelial genes was performed using Quantigene analysis (Sigma). For these applications, treatments and RNA purification were performed as described above and the subset of RNAs analyzed as denoted in Results.

### Bioinformatic identification of putative GPCRs expressed in endothelial cells

Putative GPCRs expressed in endothelial cells were identified using three database mining algorithms: 1) Genetic and eQTL association database constructed with inbred mouse genotyping and expression profiling data, searching for genes near SNPs associated with KLF2 expression; 2) Expression association database, using K nearest neighbor (KNN) algorithm searching genes with expression pattern similar to KLF2 across all samples in the database; 3) GPCRs expressed in HUVEC and HAEC cells, by searching a database compiled with both in-house and public Affymetrix (NCBI GEO database (accession number GSE2638) expression profiling data of HAEC, HUVEC and HMEC cells. Genes identified in at least 2 of the control samples across the three algorithms having the Affymetrix detection call P were selected as candidate receptors (listed in the table in Supplementary Figure S1).

### High-throughput siRNA screen

On-Target Plus^®^ siRNA Libraries were obtained from Dharmacon and were arrayed in 384 well format as 4 oligos per gene; one oligo per well. The libraries consisted of 579 GPCR sand 52 NHRs for a total of 2,524 siRNAs covering 631 genes. HUVEC cells were obtained from Lonza and reverse-transfected on a Bravo liquid handler platform. 72 h post-transfection, the KLF2 qPCR Assay was carried out using the Cells-to-C_T_ method and a duplexed TaqMan assay for KFL2 and an endogenous control gene, cyclophilin A. Hit selection criteria: average ddCt from triplicate plates, ddCt ≤ −1 or ≥ +1 (2x increase or decrease in KLF2 expression relative to that obtained with non-targeting siRNA = 1 (*p*-value ≤0.05) and ≥ Two oligos out of four per gene). The genes were ranked by the top-2 performing oligos. Using these criteria, 40 primary screening hits were retested with the same siRNAs (Dharmacon) and orthogonal siRNA sequences obtained from a different commercial source (Qiagen) and against two donors of HUVEC cells. When compared to primary screening data, 14 genes validated and were then tested in HAEC with TRC shRNA (5/gene) using multiple donors of both HUVEC and HAEC cells (Lonza).

### Lentiviral shRNA transduction

All lentiviral constructs were obtained from the Broad Institute’s RNAi Consortium (TRC). All methods for high throughput transfection quality DNA for virus production, packaging and lentiviral transductions were carried out as previously described (Moffat et al., 2006).

### SA biosciences atherosclerosis PCR array

Four million HUVECs from 2 donors (passages 3) were cultured for 2 days and then plated into 6-well collagen I plates (600K/well). The following day each of the 2 donors were transduced with 100 µL of GPR101 shRNA lentiviruses #505 and #506 (titer = 10^7^ pfu/mL) and incubated overnight. The following day, the cells were selected with 2.5 μg/mL of puromycin for 2 days after which the media was changed, and the cells incubated for an additional day. The cells were then washed with PBS, lysed, and total RNA isolated using standard procedures. The remaining steps were carried out as recommended with the Qiagen atherosclerosis RT^2^ Profiler PCR array kit (GeneGlobe ID - PAHS-038Z). Briefly, 500 ng of DNA-free total RNA was converted to first strand cDNA and diluted to a final volume of 100 µL. Real-time PCR was carried out by mixing the first strand DNA with 550 µL of RT^2^ qPCR Master Mix and diluting to 1,000 µL. The PCR array was loaded using the provided 384EZLoad Covers and the plate sealed with optical adhesive film. After centrifuging the plate at ×1500 g for 1 min, the plate as loaded into an ABI 7900HT qPCR instrument and cycled as recommended. The data were analyzed using Qiagen’s online web analysis tool to produce comparative heat maps and fold change by determining the ratio of mRNA levels to control values using the ΔCt method (2^−ΔΔCT^). All data were normalized to an average of three housekeeping genes, GAPDH, HPRT1 and β2m.

### QuantiGene plex gene expression assay

The analysis of KLF2 and related gene expression changes in response to treatments with atorvastatin and somatostatin were assessed using a custom QuantiGene Plex expression assay (Thermo Fisher Scientific) (gene IDs are provided in the Supplementary Material). After compound treatments, the cells were processed according to the manufacturers protocol and data collected on a Luminex 200 instrument. Fold-changes were calculated as the relative ratios between the normalized reference values of all treatment groups and the untreated group’s values. The data was analyzed by calculating the mean background signal, obtained from RNA free wells and subtracting it from the raw median fluorescence intensity values of RNA well samples. Relative gene expression was calculated by dividing the background corrected gene signals with the geometric mean of the housekeeping genes PPIA, GusB and HPRT. Fold changes were determined by dividing the relative expression values by the median relative expression value from the untreated negative controls.

### Endothelial barrier assay

HUVECs were seeded at 500,000 cells/well and grown to confluence on HTS Transwell-96 well membranes (Corning #3385) for 48 h. The cells were incubated with 0.4 U/mL thrombin ± compounds or DMSO for 24 h. After compound treatment, FITC-labeled dextran (1 mg/mL) was added to the upper chambers for 60 min. The medium samples were then collected from the lower chambers and fluorescence measured at 520 nm when excited at 490 nm using a SpectraMax Gemini fluorescent plate reader. Control wells (thrombin + DMSO treatment) served as the baseline permeability. The ability of compounds to prevent thrombin-induced permeability was expressed as fold change in fluorescence units from control wells.

### Monocyte adhesion assay

HUVECs were seeded at 50,000 cells/well in the upper chamber of Fluoblok insert 96 chemotaxis assay plate (BD Bioscience #351162 with 3 μm pores) for 48 h until the endothelial cells formed a confluent monolayer. On the day of assay, HUVECs were challenged with 10 ng/mL TNFα with or without compounds for 1 h. THP-1 cells were labeled with Calcein AM (Molecular Probes C3100) by mixing THP-1 cells with Calcein AM at a final concentration of 1.5 uM for 30 min. After washing the THP-1 cells twice with 200 μL culture medium, the cells were allowed to recover for at least 1 h at 37°C. Calcein-labeled THP-1 cells were then added to the upper chambers of the wells and 10 nM MCP-1 (Invitrogen #PHC1015) ± 0.1% DMSO (control) or compounds at varying concentrations added to the lower chambers. The plates were then incubated for 90 min in a cell culture incubator. The plates were read on an Envision plate reader (excitation 485 nm and emission 535 nm). Data was analyzed as fold increase compared to DMSO (agonist mode) or in response to MCP-1 over control (antagonist mode).

### MCP-1 release bioassay

HUVECs were seeded at 500,000 cells/well and grown to confluence on a 96 well plate for 48 h. The cells were incubated with 0.4 U/mL thrombin (positive control), synthetic compounds at increasing concentrations or 0.1% DMSO (negative control) for 24 h. Culture medium (50 μL/well) was then collected and MCP-1 measured in duplicate samples using a human MCP-1 ELISA Assay kit (Thermo Fisher # EH2MCP1) following manufacturing’s instructions. Data were expressed as percent of MCP-1 measured in the samples from cells treated with 0.4U/mL thrombin.

### LGR4 high-throughput screen

In order to identify novel synthetic agonists of LGR4, the BMS chemical library was screened using the DiscoveRx CHO-K1 LGR4 *ß*-arrestin cell line (Eurofins) which uses a *ß*-galactosidase enzyme complementation assay to detect ligands capable of recruiting *ß*-arrestin via LGR4. On each day of screening, HTS plates were pre-printed with library compounds (20 nL) using the ECHO acoustic dispensing system (Beckman) on an automated robotic platform. LGR4-CHO cells were then harvested and plated into the pre-printed plates (500 cells/well in 2 μL). Plates were incubated for 2 h at room temperature followed by the addition of 1 μL/well of DiscoveRx Pathhunter chemiluminescent detection reagent. Plates were incubated for an additional 1 h at room temperature and then luminescence read on a Viewlux luminescent plate reader (PerkinElmer). Compounds were considered to be active if luminescence was greater than ≥3 SD above the mean; for the LGR4 high-throughput screen using this procedure, Z’ = 0.55.

### Orbital shear assay

HUVECs were seeded at 30,000 cells per well in Collagen I coated 96 well plates as noted above. Twenty-four hours after seeding, the growth medium was replaced with serum-free medium for an additional 24 h. Cells that were previously transfected with control or targeted siRNAs, or compounds were incubated for 24–72 h prior to exposure to orbital shear. Identical cultures of cells that were not exposed to the orbital shear paradigm (static cultures) were used as additional controls. Cells were then exposed to an orbital shear force of 20 dynes/cm^2^ by shaking 96 well plates on a Thermo Fisher Scientific 96 well orbital plate shaker at 37°C in a tissue culture incubator for the designated times. Orbital shear force was estimated using the procedure of Dardik et al. (2005) and calculated using the equation, τ_max_ = $a\sqrt{\eta \rho {\left(2\pi f\right)}^{3}}$ , where τ_max_ = shear force, a = radius of rotation of the platform, η = viscosity of the culture medium, *ρ* = density of the culture medium and f = the frequency of rotation of the platform. The frequency of rotation of the shaker platform was measured and adjusted to provide the required shear force using a digital handheld laser tachometer. Following exposure to orbital shear force, the medium was collected for evaluation of released substances and the remaining cells were lysed for qRT-PCR analyses using Tryzol reagent.

Validation of the utility of the orbital shear high-throughput method was performed by comparing gene expression profiles of known laminar shear-sensitive genes to laminar shear exposed cells cultured on collagen I coated glass slides using the Flexcell STREAMER system at 20 dynes/cm^2^ for 24 h.

### Immunohistochemistry

Immunohistochemistry for LGR4 and GPR101 were performed at Lifespan Biosciences using normal human donor tissues. For LGR4, a Lifespan Biosciences rabbit polyclonal antibody, 160411- LP1631 was used, and staining patterns confirmed using a second rabbit polyclonal antibody, 160,411–3,593. In the case of GPR101, Lifespan Biosciences rabbit polyclonal antibody, 190414-LP1838 was used, and staining patterns confirmed with rabbit polyclonal antibody, 190414-LP1836. Tissue localization of receptors was independently confirmed by a pathologist at Lifespan Biosciences.

### Statistical analyses

Unless otherwise noted, all statistical analyses were performed using ANOVA along with Duncan’s multiple range test for treatment comparisons. Where variance across groups was not homogeneous, data were log-transformed prior to running statistical analyses.

## Results

### KLF2-modulating GPCRs

**FIGURE 1:**
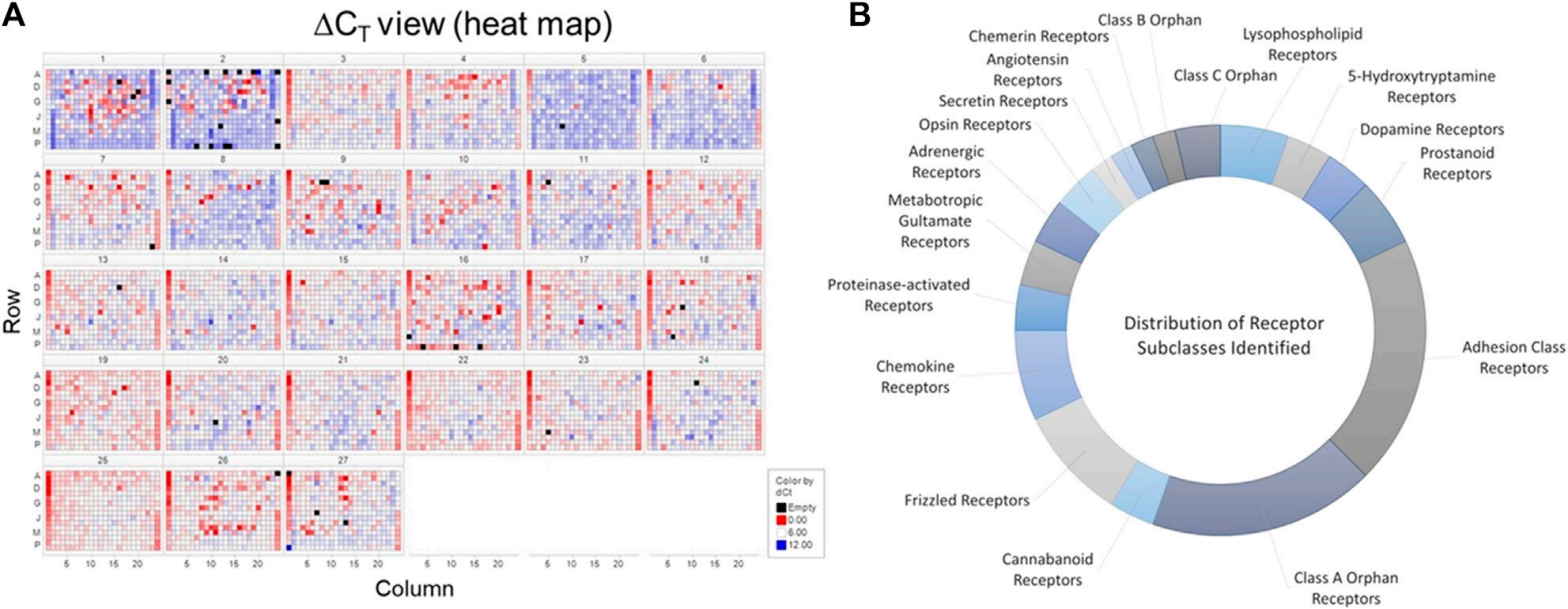
Identification of GPCRs in vascular endothelial cells. Panel **(A)** Screening results from the siRNA screen for GPCRs potentially modulating KLF2 expression in HUVECs. Data are represented in heat map form using a log2-fold change over control (GFP siRNA) treated cells. Panel **(B)**. A distribution of classes of GPCRs found to be expressed in endothelial cells. These receptors were then classified based upon IUPHAR GRAFS nomenclature (Alexander et al., 2021). The data represent the number of different receptors from each class identified in the bioinformatics analysis.

In order to identify putative GPCRs capable of affecting flow-dependent signaling within vascular endothelial cells, we employed a target screening strategy (Supplementary Figure S1A) that combined understanding of known expression of candidate GPCRs in endothelial cells using bioinformatics with validation using genetic screening utilizing siRNA. Figure 1A illustrates the heat map of the siRNA screen for GPCRs capable of modulating KLF2 expression in HUVECs. siRNAs that significantly altered normalized KLF2 expression relative to control siRNA transfected cells were identified as hits as described in *Materials and methods*; Figure 1B highlights the distribution of classes of GPCRs identified through bioinformatic analyses of in-house and external datasets. 80 receptors were identified. Among these, GPCRs with nucleotide/lipid agonists, peptide agonists, biogenic amine agonists and adhesion protein agonists were particularly well represented within these datasets (Figure 1B). In addition, there was a significant number of orphan GPCRs from Class A receptors that were found to be expressed in endothelial cells. Receptors known to be mediators of inflammatory (e.g., EP4, H1R, CXCR4/7) or thrombotic (e.g., PAR1, P2Y4/5, TBXA2R) agonists were also evident, as were several receptors of the Frizzled family. Overall, many of the classes of GPCRs found were in-line with current understanding of the role of physiological mediators in regulating endothelial function and cardiovascular pathophysiology.

**FIGURE 2:**
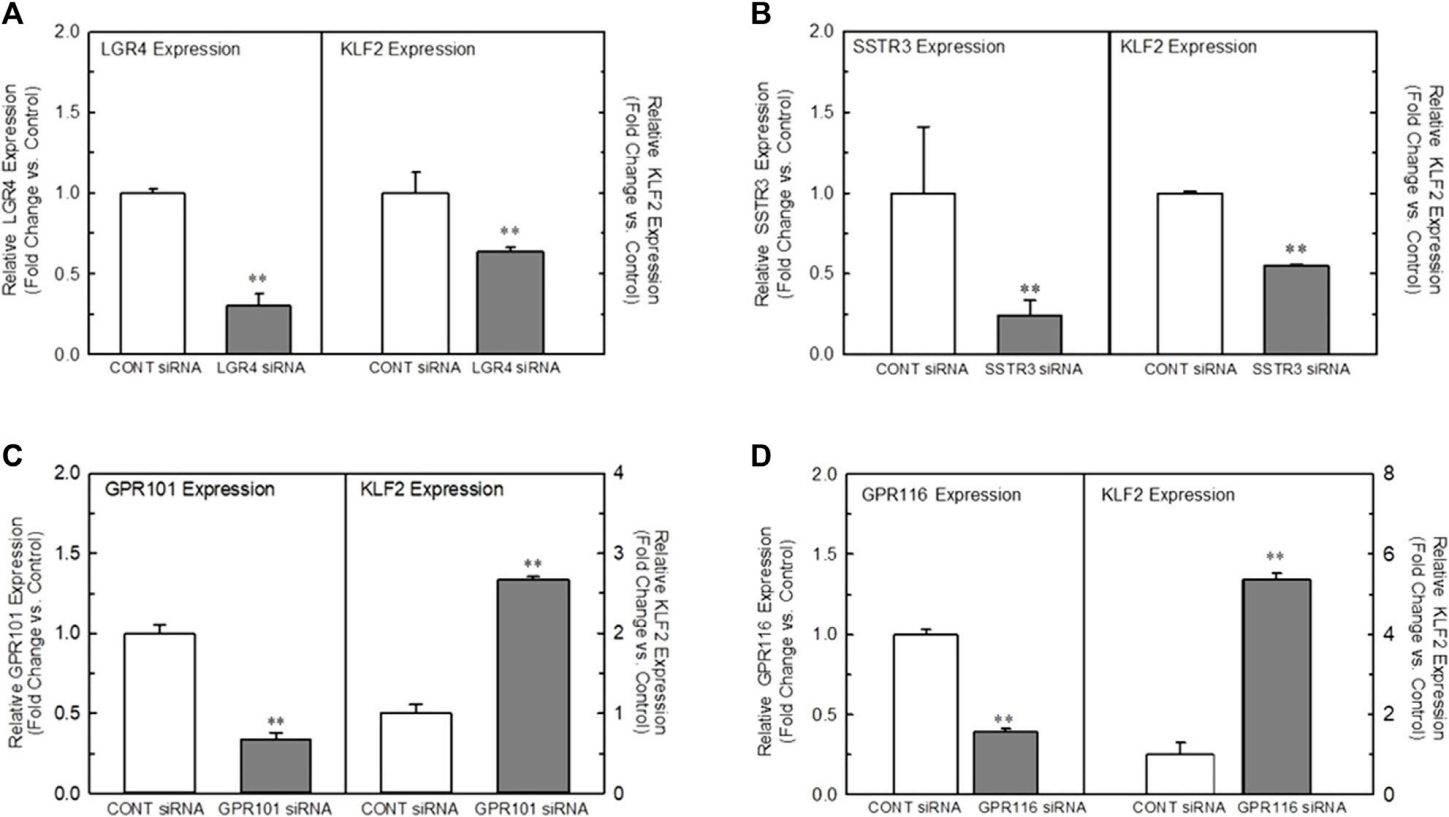
Effect of targeted knockdown of select GPCRs on endothelial KLF2 expression in static culture. Panels **(A–D)** Effect of siRNA knockdown of LGR4 Panel **(A)**, SSTR3 Panel **(B)**, GPR101 Panel **(C)**, and GPR116 Panel **(D)** on KLF2 expression in HUVECs, respectively. Endothelial cells were transfected with an siRNA targeting the indicated receptors (Dharmacon) or GFP as control. Cells were grown for 48 (LGR4, SSTR3) or 72 h (GPR101, GPR116) following transfection and then processed for determination of the target gene and KLF2 by qRT-PCR (*n* = 4). Target gene expression is illustrated on the left in each panel, while the associated KLF2 expression is depicted in the right. Control mean Ct values for target genes were: LGR4 = 26.5, SSTR3 = 33.8, GPR101 = 23.5 and GPR116 = 29.0. * = *p* < 0.05 vs. Control Group, ** = *p* < 0.01 vs. Control Group, *** = *p* < 0.001 vs. Control Group by ANOVA.

**FIGURE 3:**
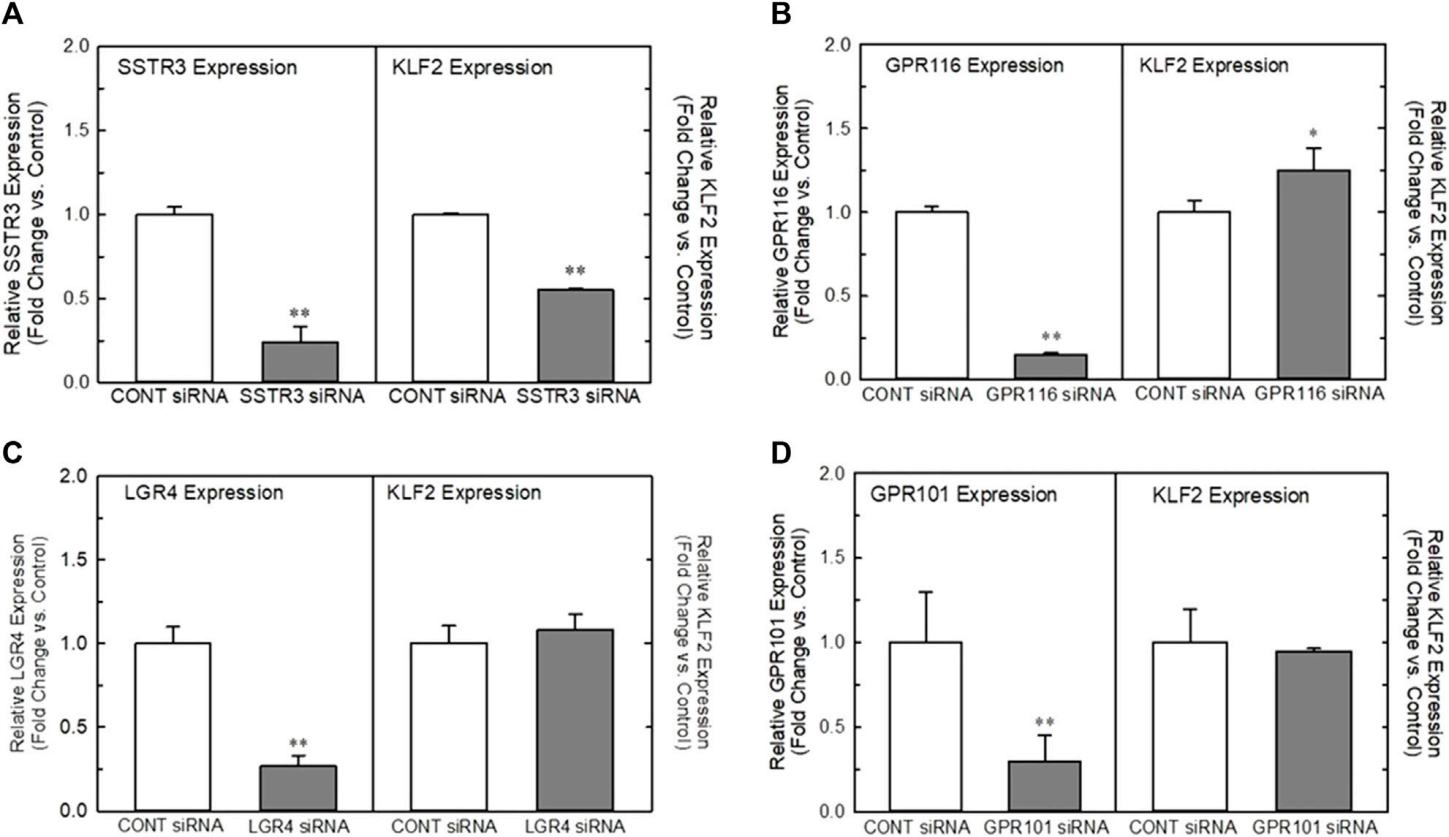
Effect of targeted knockdown of SSTR3, GPR116, LGR4, and GPR101 on KLF2 expression in endothelial cells exposed to orbital shear. Panels **(A–D)** Effect of siRNA knockdown of SSTR3 Panel **(A)**, GPR116 Panel **(B)**, LGR4 Panel **(C)**, and GPR101 Panel **(D)** on KLF2 expression in endothelial cells, respectively. Cells were transfected with an siRNA targeting the indicated receptors (Dharmacon) or GFP as control. Cells were grown for 48 (SSTR3, LGR4) or 72 h (GPR116, GPR101) following transfection and then processed for determination of the target gene and KLF2 by qRT-PCR (n = 4). Target gene expression is illustrated on the left in each panel, while the associated KLF2 expression is depicted on the right. * = *p* < 0.05 vs. Control Group, ** = *p* < 0.01 vs. Control Group, *** = *p* < 0.001 vs. Control Group by ANOVA.

In order to understand the role of the receptors identified in our analyses, we developed a high-throughput, 384-well orbital shear assay based upon the previous work of Dardik et al. (2005) that we utilized to screen the effect of receptor knockdown on flow-dependent endothelial function (Supplementary Figure S2). This system effectively reproduces gene expression profiles of laminar flow on key inflammatory and thrombotic pathway genes (Supplementary Figure S2A). In addition, it reliably reproduces the morphological changes in endothelium that occurs when they are exposed to high laminar shear forces (Supplementary Figure S2B) and stimulates KLF2 protein production (Supplementary Figure S2C). Figure 2 illustrates the impact of targeted knockdown of select receptors identified in the screening paradigm. Receptors (LGR4, SSTR3, GPR101, and GPR116) representing various classes of GPCRs were able to differentially impact KLF2 expression in primary endothelial cell cultures. In these experiments, siRNAs to each receptor or a control siRNA (GFP siRNA) were transfected into HUVEC cells and cultured 48–72 h (optimized for each target mRNA) and both receptor and KLF2 gene expression determined by qRT-PCR. Using this methodology, we were able to obtain approximately 70%–90% reduction in quantifiable mRNA expression of the receptors shown (Figures 2A–D, left panels). This level of reduction in LGR4 and SSTR3 expression significantly reduced KLF2 expression in endothelial cells in static cultures (Figures 2A, B, right panels). Conversely, knockdown of GPR101 (Figure 2C) and GPR116 (ADGRF5) (Figure 2D) both significantly elevated KLF2 expression. These data suggest that LGR4 and SSTR3 act to positively regulate KLF2 expression, while GPR101 and GPR116 act as negative regulators of KLF2 expression in endothelial cells. Furthermore, exposure of endothelial cells to orbital shear at 20 dynes/cm^2^ following knockdown of these receptors, revealed that SSTR3 (Figure 3A) and GPR116 (Figure 3B) significantly affected flow-dependent stimulation of KLF2 expression. SSTR3 KD significantly attenuated the induction of KLF2 expression by orbital shear. Conversely, reduced GPR116 expression slightly but significantly elevated KLF2 expression induced by flow. These data suggest that SSTR3 and GPR116 can potentially modulate flow-dependent regulation of KLF2 gene expression in endothelial cells. Knockdown of LGR4 (Figure 3C) and GPR101 (Figure 3D) did not significantly impact flow-dependent KLF2 expression suggesting that, although GPR101 and LGR4 can regulate KLF2 expression, they are not directly involved in the regulation of KLF2 expression induced by orbital shear.

### SSTR3 expression and regulation of KLF2 in endothelial cells

**FIGURE 4:**
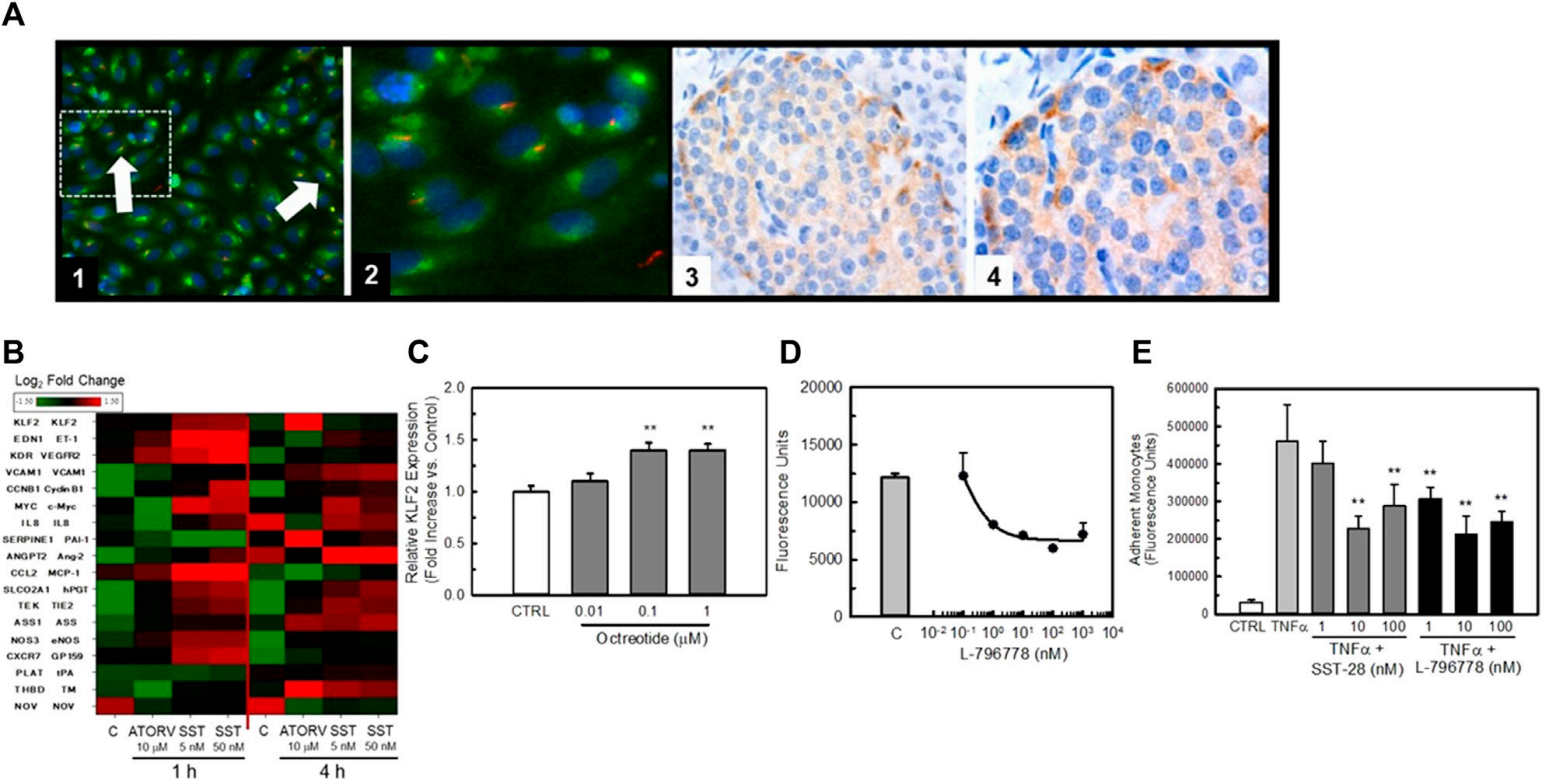
SSTR3 is present in endothelial cells and agonists positively affect KLF2 expression and improve endothelial function. Panel **(A)** Image 1, SSTR3 is co-localized with acetylated alpha-tubulin in HUVEC cells. HUVEC cells were cultured in 96 well plates for 48-72 prior to be fixed in formalin and stained with a fluorescently labeled (Texas Red) antibody to hSSTR3 and a fluorescently-labeled antibody to acetylated alpha tubulin (FITC). Co-localization of SSTR3 with acetylated alpha-tubulin (orange) is evident in HUVECs confirming the localization of SSTR3 to primary cilia in HUVEC cells. (Magnification ×200). An area of interest (dashed line) from this image is expanded in Image 2. Images 3 and 4, confirmation of SSTR3 expression in pancreas from normal human tissue. Panel **(B)** Heat map of expression of endothelial genes following exposure to either 5 μM atorvastatin (positive control) or increasing concentrations of SST28 for either 1 or 4 h expressed as Log2-fold change over untreated controls. Gene names and their respective mature protein sequences are indicated on the left side of the heat map. Panel **(C)** The SSTR3 partial agonist, octreotide, dose-dependently increases KLF2 expression in HUVEC cells. Treatment of HUVEC cells for 4 h with octreotide significantly increased KLF2 expression (*n* = 4), ** = *p* < 0.01 vs. Control Group by ANOVA. Panel **(D)** The SSTR3-selective agonist L-796778 dose-dependently improves barrier function in HUVEC cells. HUVECs were grown to confluence in 96 well transwell plates before being exposed to thrombin ± increasing concentrations of L-796778 (*n* = 8). L-796778 dose-dependently reduced HUVEC permeability to FITC-dextran. Panel **(E)** SSTR3 agonists improve monocyte adhesion to HUVEC cells. HUVECs were grown to confluence in 96 well transwell plates. Cells were then treated with 10 ng/mL TNFα ± increasing concentrations of SST-28 or L-796778. THP-1 cells were loaded with Calcein AM prior to addition to the transwells and incubated for 4 h. Wells were then washed and fluorescence measured. Both SST28 and L-796778 dose-dependently decreased THP-1 cell adhesion to HUVEC cells (*n* = 4). ** = *p* < 0.01 vs. Control, *** = *p* < 0.001 vs. either control siRNA or TNFα only treated cells by ANOVA.

Figure 4 illustrates the localization of SSTR3 in endothelial cells and the effect of agonists and antagonists to modulate KLF2 expression. Confocal microscopy using a high content imaging system (Cellomics) of HUVECs for immunoreactive SSTR3 revealed strong immunofluorescence of SSTR3 in HUVECs (Figure 4 Panels 1 and 2, green). Co-staining of the same cells with an antibody to acetylated-α-tubulin (Figure 4A Panels 1 and 2, red, a marker of the primary cilium) confirmed the presence of primary cilia on HUVECs in culture. The presence of co-staining for acetylated *α*-tubulin and SSTR3 demonstrated co-localization of SSTR3 and acetylated *α*-tubulin (Figure 4A Panels 1 and 2, orange) suggesting localization of SSTR3 to the primary cilium in primary cultures of endothelial cells, a known mechano-transducing organelle. As reported by Shahbaz et al. (2015), immunohistochemistry of human pancreatic tissues identified specific SSTR3 immunostaining in pancreatic islets (Figure 4, Panels 3 and 4, brown) and in the endothelium of pancreatic vessels.

In order to study the impact of SSTR3 activation on HUVEC gene expression we challenged HUVECs with the naturally occurring agonist for SSTRs, somatostatin (somatostatin 1–28 (SST)), for 1 or 4 h (Figure 4B). We then quantified expression of KLF2 and known KLF2 target genes using Quantigene analysis. As can be seen in the heat map, SST rapidly stimulated KLF2 gene expression within 1 h of treatment. The positive control, atorvastatin (ATORV) (Parmar et al., 2005), also stimulated KLF2 expression but this was delayed in onset as compared to SST. Both SST and ATORV induced a gene-expression profile consistent with a positive role for SSTR3 in modulating KLF2 gene expression (e.g., elevation in thrombomodulin (TM) and eNOS, reduction in tissue plasminogen activator (tPA) and PAI-1). In order to confirm the ability of SSTR3 activation to stimulate KLF2 expression, we exposed HUVECs to the SST analog, Octreotide, for 2 h and quantified KLF2 expression by qRT-PCR. As is shown in Figure 4C, octreotide dose-dependently stimulated KLF2 expression albeit modestly. Furthermore, the SSTR3 selective partial agonist, L-796778, dose-dependently improved barrier function (Figure 4D) and TNFα-induced monocyte adhesion (Figure 4E) to HUVEC monolayers, confirming the anti-inflammatory activity of SSTR3 activation consistent with a stimulation of the KLF2 pathway.

### Validation of a novel orphan GPCR in regulation of flow-mediated KLF2 expression

**FIGURE 5:**
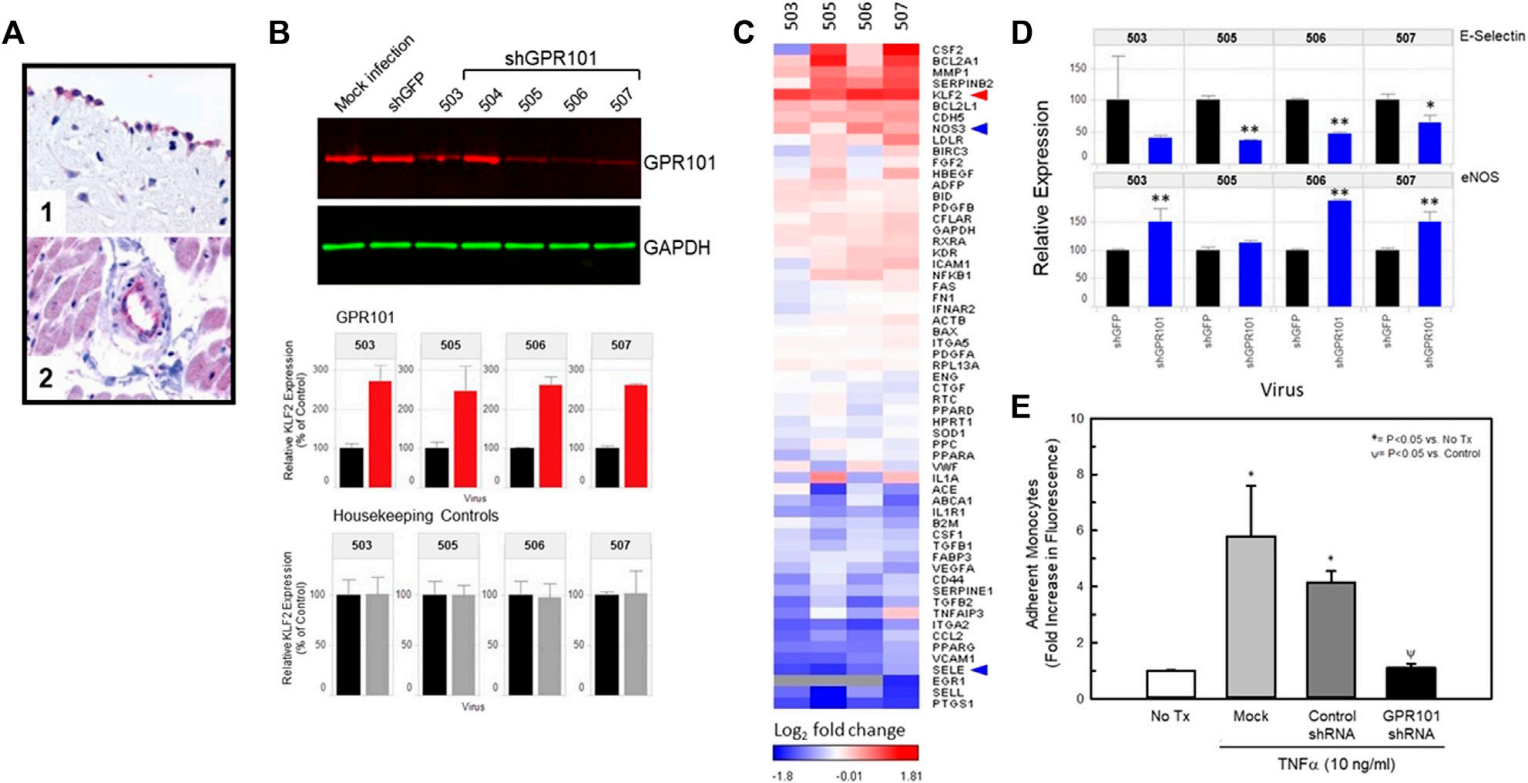
Knockdown of the class A orphan receptor GPR101 positively affects KLF2 expression and improves endothelial function. Panel **(A)** Immunohistochemistry for GPR101 immunoreactivity in normal, human vascular tissues. Localization of GPR101 immunoreactivity in endothelial cells of aorta (1) and cardiac capillaries (2). Panel **(B)** Viral delivery of shRNAs to GPR101 significantly reduce GPR101 expression in HUVECs by Western analysis (top) and concomitantly elevate endothelial cell KLF2 expression (middle) relative to GAPDH (bottom). Panel **(C)**, Heat map of the effect of KD of GPR101 in HUVECs on KLF2 and known KLF2 target genes expressed as compared to shRNA controls (GFP shRNA). GPR101 KD promotes a KLF2-like transcriptional profile that elevates KLF2 expression and eNOS, while reducing E-selectin and VCAM. Panel **(D)**, further detail of the impact of GPR101 KD on eNOS and E-selectin expression in HUVECs. E-selectin (top) expression was reduced by all of the shRNAs studied. However, shRNAs, 503, 506 and 507 all significantly elevated eNOS relative to the shRNA to GFP (control) expression while shRNA 505 did not affect eNOS expression. Panel **(E)**, KD of GPR101 improves endothelial function. KD of GPR101 with shRNA 506 significantly reduced TNFα-induced monocyte adhesion to HUVECs in culture relative to uninfected and control shRNA (GFP shRNA) infected cells by ANOVA.

One of the targets identified in our screen was the orphan receptor, GPR101. This receptor is detectable in endothelial cells of normal human vascular tissues (Figure 5A, Panels 1 and 2). Figure 5B illustrates the validation of this orphan receptor as a KLF2 modulating receptor in endothelial cells. shRNAs (Dharmacon) to GPR101 were delivered by viral delivery to HUVECs and their effect on HUVEC gene expression studied 72 h post-infection. Figure 5B (top panel) illustrates the relative effect of GPR101 knockdown on GPR101 protein abundance in HUVECs as measured by Western blot. The ability of each shRNA to reduce GPR101 protein abundance in HUVECs varied with shRNAs 505, 506, and 507 achieving the most effective knockdown. shRNA 504 did not significantly lower GPR101 protein abundance, therefore, subsequent studies utilized shRNAs 503, 505, 506, and 507. Measurement of KLF2 expression in the same cells, revealed that shRNAs 503, 505, 506, and 507 significantly elevated KLF2 expression by 2-3-fold confirming the ability of GPR101 knockdown to positively modulate KLF2 expression. Figure 5C illustrates the impact of knockdown of GPR101 on key endothelial genes. KLF2 expression was elevated in the presence of the 4 shRNAs studied. In addition, there was notable elevation in pro-survival, anti-inflammatory and anti-thrombotic genes such as, NOS3, SOD1, PPARA, RXRA, BCL2A1, and CDH5. A concomitant decrease in pro-inflammatory and pro-thrombotic genes such as, CCL2, EGR1, VCAM1, PTGS1, vWF, SERPINE1, and SELE, was also evident. These data suggest that knockdown of GPR101 in endothelial cells induces an anti-inflammatory and anti-thrombotic transcriptional program. Figure 5D illustrates in greater detail the impact of the shRNAs to GPR101 on E-selectin and eNOS. The beneficial impact of this transcriptional program on endothelial function is represented in Figure 5E where knockdown of GPR101 resulted in a complete reversal of TNFα-induced monocyte adhesion.

### LGR4/GPR48 modulates the KLF2 transcriptional program

**FIGURE 6:**
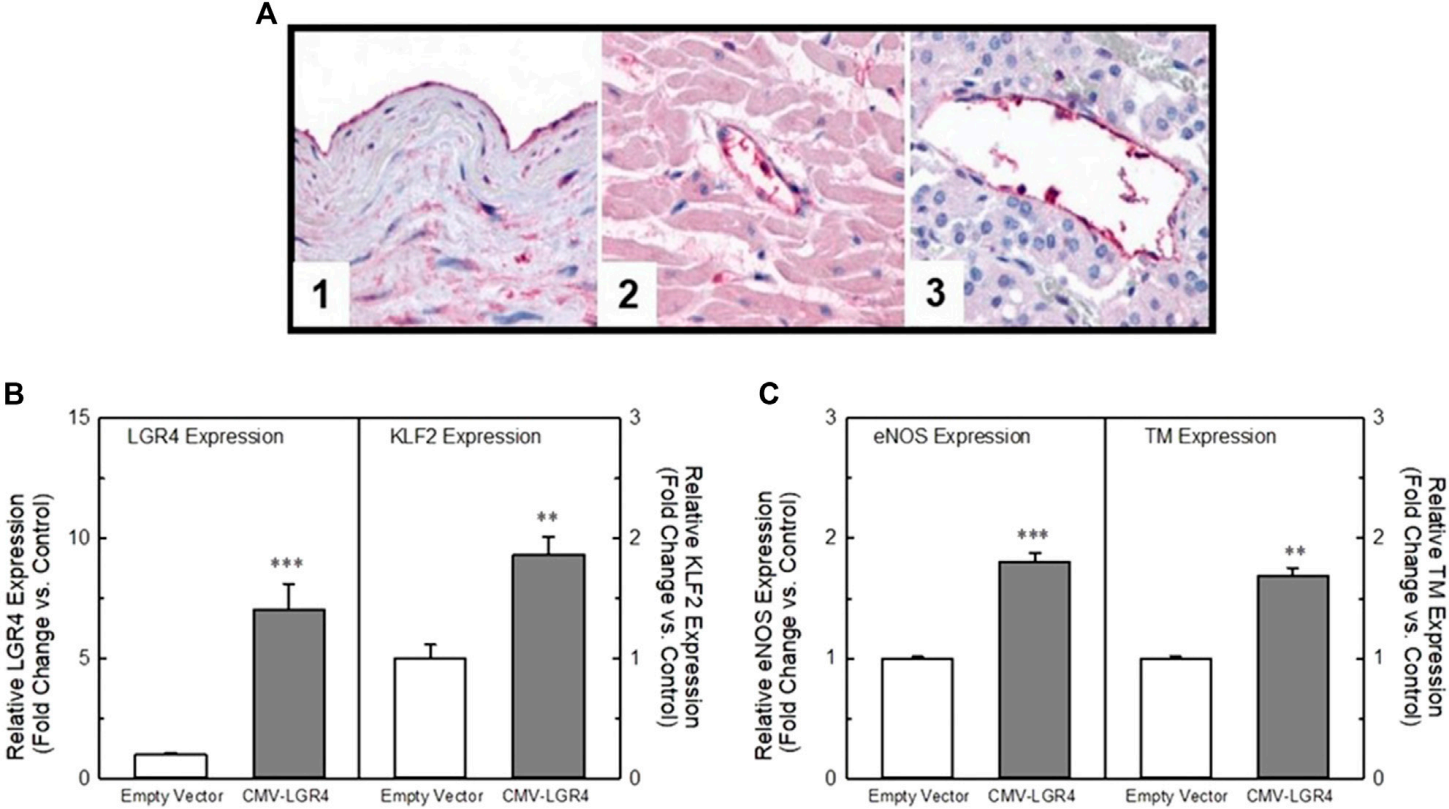
LGR4 is expressed in endothelial cells in a flow-dependent manner and can modulate KLF2 expression. Panel **(A)** Immunohistochemistry of LGR4 in normal human vascular tissue. LGR4 immunoreactivity was evident in endothelium, elastic lamina and smooth muscle of arterial tissue **(1)**. In addition, LGR4 immunoreactivity was evident in the endothelial cells of capillaries of many tissues including the heart **(2)** and adrenal gland **(3)**. Panel **(B)** Overexpression of LGR4 increases KLF2 expression in HUVECs. HUVECs were transfected with a plasmid containing the hLGR4 sequence under the control of a CMV promoter. The plasmid significantly increased expression of LGR4 (left panel). In the same cells, KLF2 expression (right panel) was significantly elevated relative to the empty vector control. Panel **(C)** Overexpression of LGR4 concomitantly elevated both eNOS (left panel) and thrombomodulin (right panel) (known KLF2 target genes) expression to a degree similar to KLF2. n = 4, ** = *p* < 0.01 vs. vector control, *** = *p* < 0.001 vs. vector control by ANOVA.

We identified LGR4 (LGR4/GPR48) as a KLF2 modulating GPCR in our bioinformatics screen (Figure 1). Although KD of this receptor did not impact flow-dependent KLF2 activation in endothelial cells, we looked further into the effect of LGR4 on endothelial gene expression. LGR4 IHC found LGR4 immunoreactivity evident in normal human vascular tissue samples (Figure 6A, Panels 1–3) where LGR4 was localized to endothelial cells in aorta (Figure 6A, Panel 1) as well as capillaries in many tissues including in the heart (Figure 6, Panel 2) and adrenal (Figure 6, Panel 3). In order to understand the impact of shear force on LGR4 expression in HUVECs in culture, we exposed HUVECs to orbital shear at 20 dynes/cm^2^ for varying amounts of time and measured both LGR4 and KLF2 expression by qPCR (Supplementary Figure S3). Exposure of HUVECs to orbital shear increased both LGR4 and KLF2 expression in a time-dependent manner. Fluid shear increased LGR4 expression within 24 h after which receptor expression declined toward basal even in the face of continued fluid shear. In the same cells, KLF2 expression was rapidly elevated within 2 h and peaked with 24 h of exposure to fluid shear. Control cultures of HUVECs (static culture) did not show changes in LGR4 or KLF2 expression over the 48 h of the experiment. In order to confirm the impact of LGR4 expression on KLF2 expression, we overexpressed LGR4 in HUVECs using a plasmid containing the hLGR4 sequence under the control of the CMV promoter. Transfection of the hLGR4 expression plasmid in HUVECs led to a 7-fold increase in LGR4 expression as measured by qRT-PCR (Figure 6B, left panel) as compared to cells transfected with the empty expression vector. Elevation in LGR4 expression was associated with a concomitant 2-fold increase in KLF2 expression (Figure 6B, right panel). In addition, elevation of LGR4 expression also induced an approximate 2-fold increase in KLF2 target genes, eNOS and thrombomodulin (TM) (Figure 6C, left and right panels, respectively). Overexpression of LGR4 in EA-hy926 cells also resulted in a significant elevation in KLF2 promoter-driven luciferase activity (Supplementary Figure S4). Taken together, these data suggest that LGR4 expression is associated with a positive regulation of KLF2 expression in HUVECs independently from the effects of fluid shear force.

### Identification of synthetic agonists to LGR4 as proof-of-concept for drug discovery of an orphan GPCR

**FIGURE 7:**
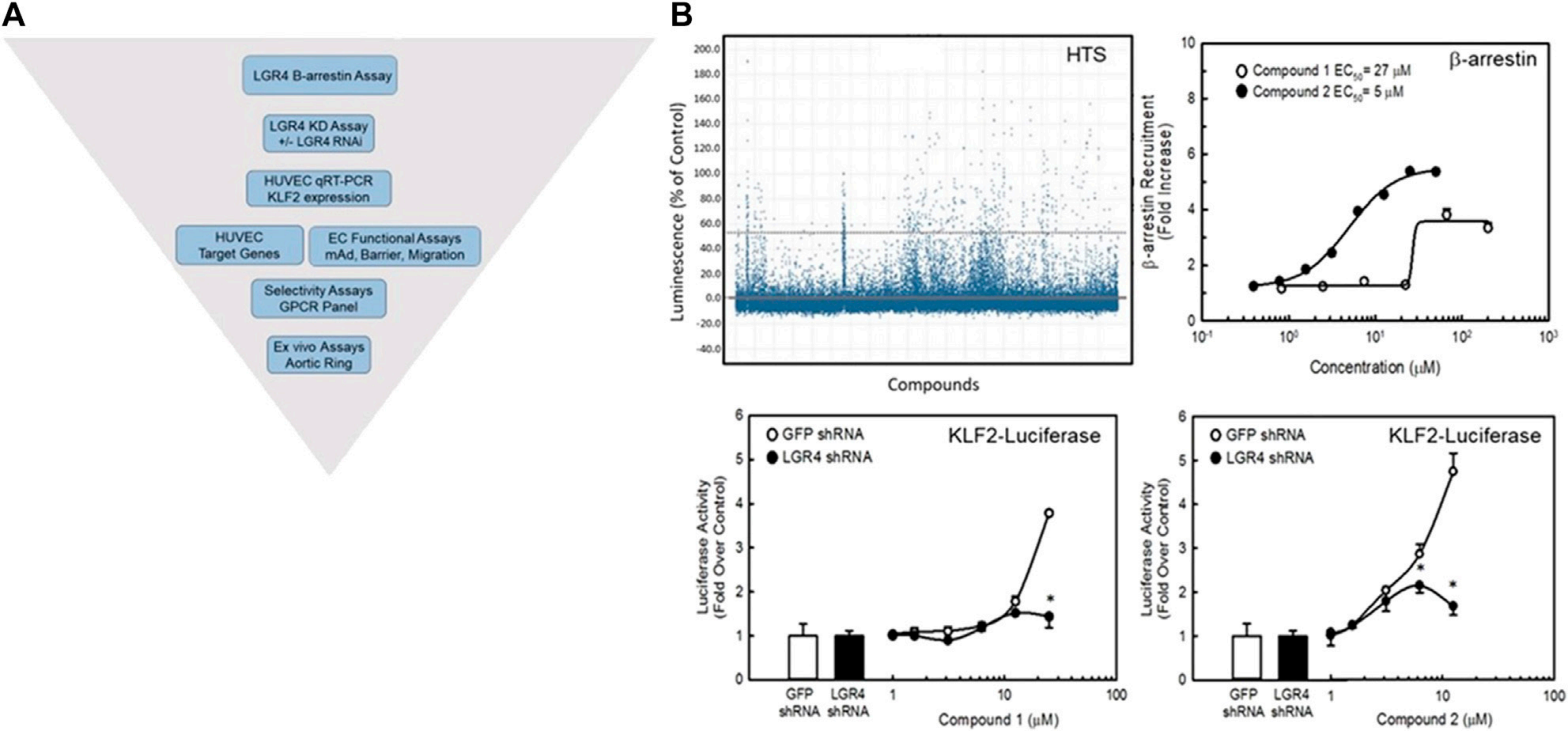
Identification of small molecule agonists to LGR4. Panel **(A)** A diagrammatic representation of the screening strategy used to identify potential small molecule agonists of LGR4. An enzyme complementation assay for *ß*-arrestin recruitment was employed as the primary screen of a library of approximately 1.2 million compounds from the BMS compound library in CHO cells overexpressing the LGR4 receptor. Compounds were assessed for requirement of LGR4 expression using a KLF2-luciferase reporter cell line overexpressing LGR4 in EA-hy926 cells in the presence of control (GFP) or LGR4 shRNA. Functional assessment of compound activities was obtained in primary HUVECs and endothelial cell functional assays. Panel **(B)** Screen results and activities of two representative compounds (Compound 1 and Compound 2) identified in the HTS. Top left, summary data for HTS using the *ß*-arrestin enzyme complementation assay. A cut-off of 3 SD was used to identify hits (points above top dashed line). Top right, Compound 1 and Compound 2 both dose-dependently recruited *ß*-arrestin. Bottom panels, KD of LGR4 inhibited the ability of these compounds to stimulate a KLF2-luciferase reporter gene in EA.hy926 cells. n = 4, * = *p* < 0.05 vs. GFP shRNA Control by ANOVA.

**FIGURE 8:**
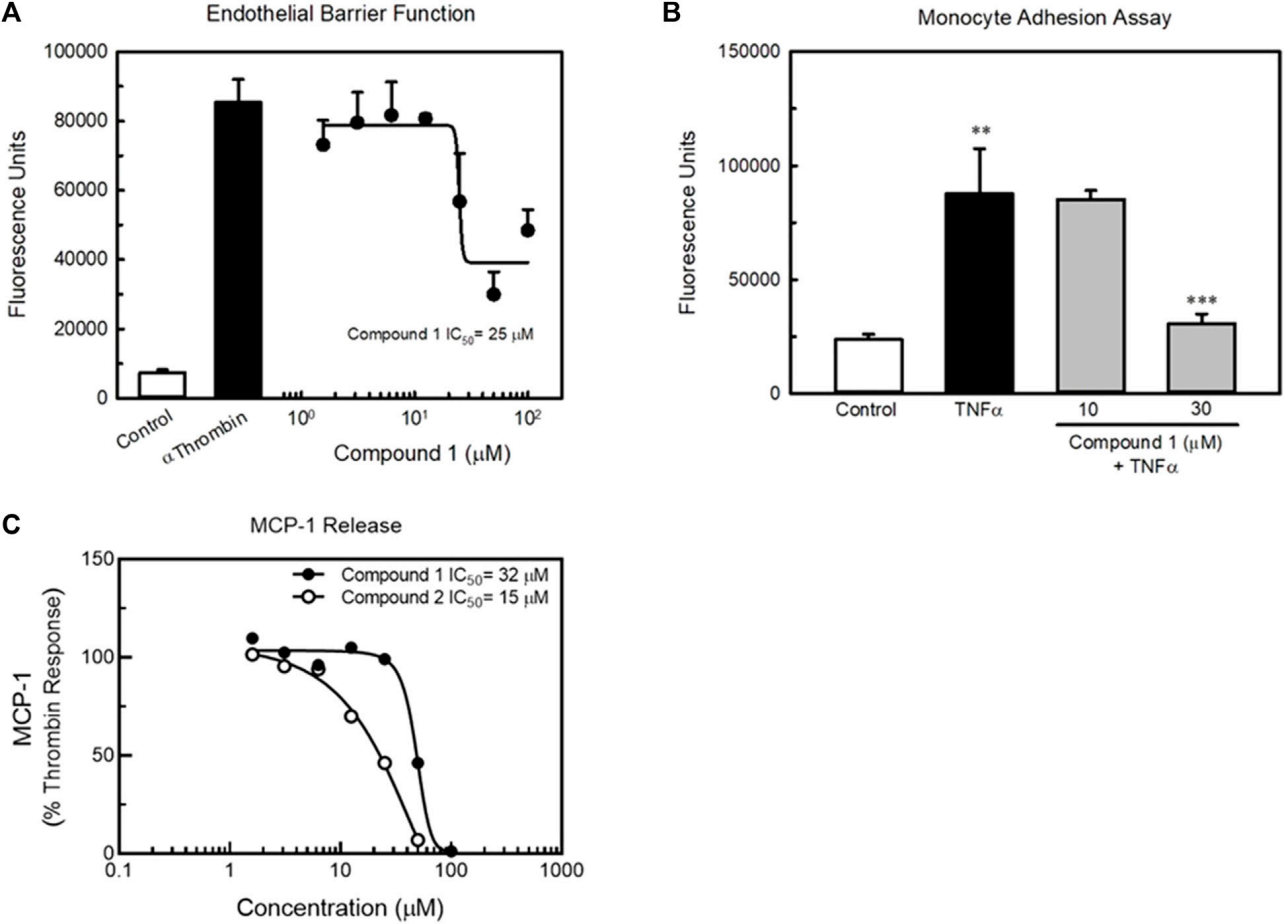
Compound 1 and Compound 2 improve endothelial cell function *in vitro*. Panel **(A)** Compound 1 dose-dependently improved endothelial barrier function of HUVECs in culture as determined by permeability of a HUVEC monolayer to FITC-dextran (top left). The potency of Compound 1 to improve barrier function was similar to its potency to recruit *ß*-arrestin. Panel **(B)** Compound 1 inhibits TNFα-induced monocyte adhesion in HUVECs. Panel **(C)** Compound 1 and Compound 2 dose-dependently reduced thrombin-induced MCP-1 release by HUVECs *in vitro*. *n* = 4, ** = *p* < 0.01 vs. Control, *** = *p* < 0.001 vs. TNFα-treated cells by ANOVA.

In order to confirm the utility of our approach targeting GPCRs to beneficially modulate endothelial function via the KLF2 pathway, we developed a high-throughput screening strategy for identifying small molecule agonists of LGR4 (Figure 7A). We miniaturized and automated a commercially available *ß*-arrestin enzyme complementation assay (DiscoveRx/Eurofins) to screen compounds from the BMS compound collection (approximately 1.2 million compounds) in 1,536 well format (Figure 7B top left panel, Z’>0.5). An unrelated GPCR expressing cell line was used as a control to identify false positive compounds. The HTS identified approximately 14,000 compounds that increased *ß*-arrestin recruitment in the LGR4-expressing cells but not in the counter-screen cell line. Of this subset of compounds, 1,154 compounds met criteria of confirmed hits following dose-response experiments. Structures of two representative confirmed hits are shown in Supplementary Figure S5. Dose-response curves for these compounds to stimulate *ß*-arrestin recruitment is also shown (Figure 7B, top right panel). Compound 1 (EC_50_ = 27 μM, 3-fold increase) was significantly weaker in potency and efficacy relative to Compound 2 (EC_50_ = 5 μM, 6-fold increase). Furthermore, both compounds dose-dependently increased activity in a luciferase reporter assay (EA-hy926 cells) under the control of the KLF2 promoter (Figure 7B, lower panels, white symbols). In order to demonstrate the requirement of LGR4 for the activity of these compounds, KD of LGR4 in this assay resulted in complete loss of activity (Figure 7B, lower panels, black symbols). Study of Compound 1 and Compound 2 in endothelial functional assays revealed that Compound 1 dose-dependently improved endothelial barrier function in the presence of thrombin (Figure 8A) and TNF-α- induced monocyte adhesion (Figure 8B). Furthermore, both Compound 1 and Compound 2 completely blocked thrombin-induced MCP-1 release with potencies in-line with their activity in the *ß*-arrestin and luciferase reporter assays (Figure 8C).

### Effects of LGR4 agonists is not through the canonical Wnt signaling pathway

**FIGURE 9:**
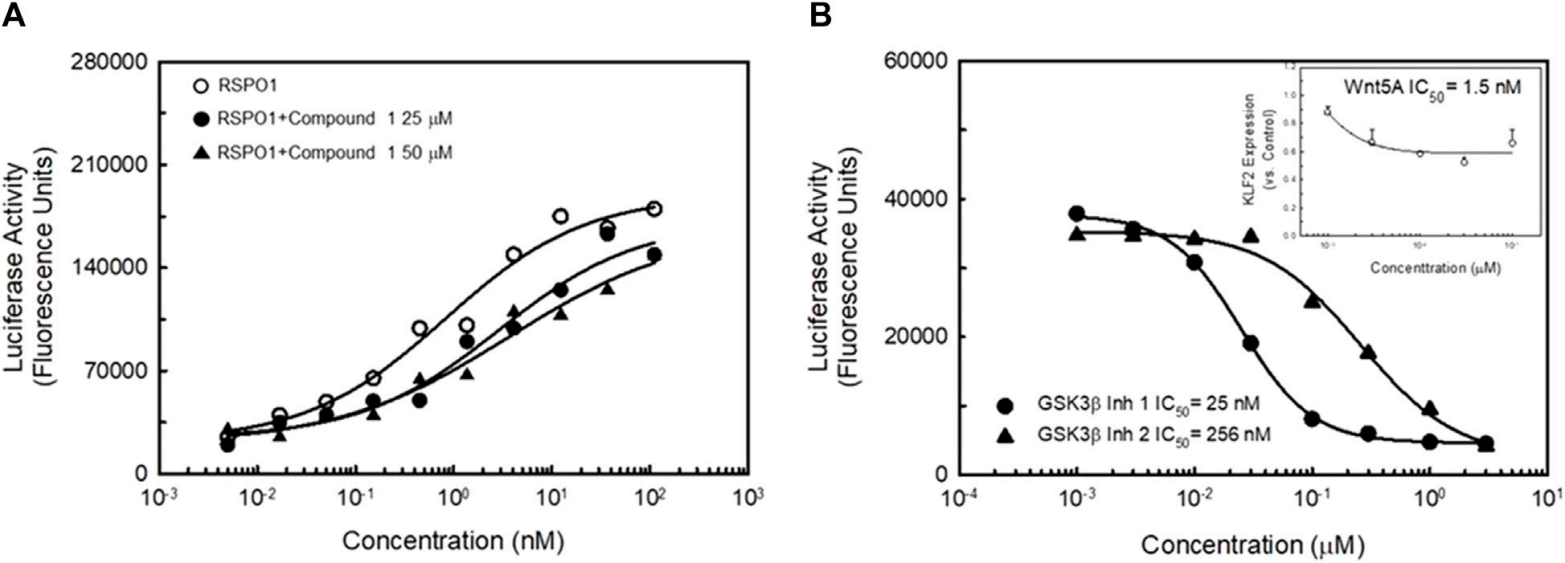
LGR4 synthetic agonists do not activate the Canonical Wnt signaling pathway. Panel **(A)** HEK923 cells co-expressing LGR4 and a TCF-luciferase reporter were incubated with either RSPO1 or RSPO1 + Compound 1. Activation of the canonical Wnt signaling pathway was determined by activation of the TCF-luciferase reporter. RSPO1 dose-dependently stimulated TCF-luciferase activity. This effect was not further enhanced by exposure of cells to either 25 μM (EC_50_ in KLF2 luciferase assay) or 50 μM (EC_80_ in KLF2 luciferase assay) Compound 1. Compound 1 induced a slight reduction in efficacy and potency of RSPO1 to stimulate the TCF-reporter. Panel **(B)** GSK3β inhibitors dose-dependently reduce KLF2-luciferase activity. Two known GSK3β inhibitors dose-dependently inhibited KLF2-luciferase reporter activity. In addition, Wnt5A (a potent agonist of canonical *ß*-catenin signaling) potently inhibited KLF2 expression in HUVECs (inset). *n* = 4.

Since LGR4 has been shown to act in concert with Frizzled receptors and LRPs to activate the canonical Wnt signaling pathway, we studied the ability of Compound 1 to modulate the effects of R-spondin 1 (RSPO1) in a TCF-luciferase assay. RSPO1 has been identified as a natural ligand for LGR4 (Carmon et al., 2011; Kang et al., 2016). RSPO1 potently stimulated TCF-luciferase activity (Figure 9A). Adding Compound 1 to the RSPO1 dose-response did not affect TCF-luciferase activity significantly. However, addition of Compound 1 did slightly right-shift the RSPO dose-response and slightly decreased the apparent efficacy of RSPO1 in the assay suggesting that Compound 1 weakly antagonized the effect of RSPO1. In contrast, in-house identified GSK3β inhibitors (Sivaprakasam et al., 2015) both dose-dependently inhibited TCF-luciferase activity (Figure 9B, black symbols). Lastly, addition of Wnt5A to the KLF2-luciferase assay resulted in a dose-dependent decrease in KLF2 expression (Figure 9B, inset) suggesting that activation of the canonical Wnt signaling pathway in endothelial cells produces the opposite effect on KLF2 expression as LGR4 agonists.

## Discussion

Cardiovascular disease remains the primary cause of mortality throughout the developed world. Existing clinical therapies taken together with diet and exercise, only partially protect against the progression of cardiovascular disease and there remains a significant unmet need for additional clinical tools. Therefore, identification of druggable new targets is an important goal toward finding new and more effective therapies to treat and prevent cardiovascular disease. The endothelium represents a key regulator of cardiovascular health given its anatomical association with flowing blood and physiological role in regulating blood pressure, perfusion, inflammation, vascular permeability and thrombosis (Alexander et al., 2020). As a result, the endothelium provides an important opportunity for developing new therapies to combat cardiovascular disease.

Indeed, the endothelium represents a key point of regulation of the cardiovascular system that extends well beyond its role as a semipermeable barrier (Alexander et al., 2020). Through synthesis and release of vasoactive substances (nitric oxide and PGI2, among others), the endothelium regulates vasoconstriction and inflammatory processes that protect from the development of atherosclerosis and platelet activation (Cai and Harrison, 2000; Sun et al., 2019). Many factors associated with increased cardiovascular risk have been found to induce endothelial damage leading to endothelial activation and dysfunction (Mundi et al., 2018). Many of these factors are also associated with inflammation and activation of endothelium. Inflammatory cytokines such as IL1β have direct effects on endothelial function (Puhlmann et al., 2005; Theofilis et al., 2021) and there is a direct correlation between cardiovascular health and endothelial phenotype (Widlansky et al., 2003). These mediators lead to loss in barrier function of the endothelium as well as an increased ability of monocytes to adhere to and transmigrate through the endothelium and promote inflammation as part of atherosclerotic pathogenesis (Raggi et al., 2018).

A primary regulator of anti-inflammatory and anti-thrombotic effects of the endothelium is the pattern and shear force of flowing blood over its surface. Decades of research by many have demonstrated the anti-inflammatory and anti-thrombotic phenotype induced by high (>12 dynes/cm^2^), laminar fluid shear force and the proinflammatory, pro-thrombotic phenotype induced by low (<5 dynes/cm^2^), oscillating or turbulent fluid shear force on endothelial cells. These effects are evident in the morphology, barrier function, monocyte adhesion, release of bioactive mediators and gene expression patterns within these cells (for review see (Traub and Berk, 1998; Zhou et al., 2014; Roux et al., 2020)). This provides an intriguing opportunity to design specific positive regulators of this important physiological phenomenon through identification of proteins and pathways capable of responding to, or modulating flow.

In the series of studies presented here, we have described an approach for identifying drug discovery targets that modulate flow-dependent effects in endothelial cells. Our approach relied upon the well described effector of laminar flow, the KLF2 transcription factor. The role of KLF2 in mediating the positive effects of laminar shear is well described (SenBanerjee et al., 2004; Parmar et al., 2005; Parmar et al., 2006; Roux et al., 2020). Understanding how the endothelium perceives the stimulus of laminar flow to activate the KLF2 transcriptional program is less well understood. In our studies, we looked to understand if GPCRs were capable of modulating KLF2 expression in endothelial cells and to understand if they were potentially mechano-transducers acting as receptors of physical force at the cell surface. A number of GPCRs have been reported to be mechano-transducers including AT1R (Mederosy Schnitzler et al., 2008; Wang et al., 2018), GPR56 (White et al., 2014) and GPR68 (Xu et al., 2018). In these studies, we sought to take a comprehensive review of GPCRs within endothelial cells. The objective was to understand if any of these receptors were capable of affecting or mediating the effects of laminar shear on KLF2 transcriptional activation and thus would represent novel druggable targets for the development of therapeutics.

Using a multi-phase approach that integrated bioinformatics and RNAi screening, we identified numerous GPCRs capable of modulating KLF2 expression in endothelial cells. These receptors were representative of many classes and sub-families of GPCRs including adhesion receptors (represented by GPR116), Class A receptors (SSTR3) and members of the LGR family (LGR4), among others. Of particular interest were several orphan receptors including GPR101 that demonstrated robust activity in modulating KLF2 expression. Recently, Kaur et al. have studied classes of GPCRs present and expressed in murine vascular cell types, including endothelial cells, using a single-cell gene expression approach (Kaur et al., 2017). Of particular interest to the data presented here, they studied the receptors expressed from cells associated with different vascular beds and observed that the expression patterns were heterogeneous across the tissues studied. This is in agreement with known pleiotropic genotypes of endothelial cells from different vascular beds (Chi et al., 2003; Jambusaria et al., 2020) that likely give rise to tissue-specificity of function. Comparison of the receptors expressed in their study with those described here in human cells, reveals striking similarities in terms of the receptors identified. Notable examples of overlap include: LGR4, GPR116, GPR56, GPR137, S1P1R, and GPRC5 family members. Kaur et al. (2017) noted significant changes in expression of these receptors depending upon the tissue derived. To the extent there is significant species differences between the two studies as well as methodological differences (our studies utilized pooled endothelial cells from multiple human donors) it is difficult to draw a direct correlation between the two datasets. However, despite the obvious differences between the two studies, we find significant overlap in many of the receptors identified by the two approaches.

In our studies, targeted KD of these receptors either reduced or stimulated KLF2 expression in endothelial cells and, in some cases, KD was capable of impacting flow dependent KLF2 expression *in vitro*. Importantly, adhesion receptors such as GPR116 (ADGRF5) and GPR56 were observed as it was hypothesized that the process would potentially observe this class of GPCRs as being present in endothelial cells and capable of impacting KLF2 expression. Due to their proximity to the extracellular matrix and structural motifs capable of interacting with the extracellular matrix, these GPCRs had been hypothesized to have potential as mechano-transducers capable of perceiving mechanical force that impinges upon the endothelial glycocalyx. Here we report for the first time that GPR116 is capable of modulating the effects of laminar flow on endothelial transcriptional programming. Interestingly, KD of GPR116 (ADGRF5) was associated with an increase in KLF2 expression, suggesting GPR116 elicits a tonic inhibitory effect on KLF2 expression. This is an interesting observation in light of recent data suggesting proinflammatory effects of constitutive KO of GPR116 in mice (Fukuzawa et al., 2013; Kubo et al., 2019) and anti-inflammatory effects of a putative ligand for GPR116, FDNC4, in macrophages (Bosma et al., 2016; Georgiadi et al., 2021). The reasons for the discrepancy between these observations and those presented here may be due to differential activation of GPR116 in the presence of FDNC4 versus laminar shear. Similarly, GPR116 may have different roles in the endothelium than in other cell types. Indeed, GPR116 KO mice display significant vascular leakage suggesting a protective role of GPR116 in the endothelium (Niaudet et al., 2015; Zaidman et al., 2020). Additional studies are needed to further understand the role of GPR116 as a mechano-transducer/modulator of endothelial cell function.

In contrast to GPR116, SSTR3 KD was associated with a reduction in basal and flow dependent KLF2 expression in endothelial cells. Unlike, GPR116, SSTR3 provided an easier path to target confirmation/validation due to the numerous pharmacological and other tools available for interrogating this receptor. Indeed, known SSTR3 agonists (SST28, Octreotide and L-796778) replicated the biological effects expected by inducing KLF2 expression and modulating known KLF2 target genes. SSTR3 agonists induced an anti-inflammatory, anti-thrombotic profile in endothelial cells and this was reflected in improved barrier function and reduced monocyte adhesion. That SSTR3 may be responding to the force of fluid flow over the surface of the endothelium is further bolstered by the well documented localization of this receptor to the primary cilium. The primary cilium is a non-motile cilium that responds to mechanical force by inducing signaling pathways intracellularly (Spasic and Jacobs, 2017). The primary cilium has been implicated in the regulation of endothelial function and as a key mediator of flow dependent KLF2 induction (Hierck et al., 2008; Li et al., 2020) and is a known node for signaling especially as it relates to cell cycle regulation (Doxsey et al., 2005; Wachten and Mick, 2021). Both SSTR3 and 5HTR6 have been found to contain ciliary localization sequences directing their expression to the surface of the primary cilium (Berbari et al., 2008; Chadha et al., 2021). In our studies, immunofluorescence staining of HUVECs in culture detected co-localization of SSTR3 and the ciliary marker, acetylated *α*-tubulin, confirming ciliary localization of SSTR3 in endothelial cells. We also noted that not all cells in culture had a detectable primary cilium. This is likely due to cell cycle regulation of the primary cilium since resorption of the primary cilium has been proposed to be an important step in cells progressing from G2 to M phase of the cell cycle (Guo et al., 2007; Plotnikova et al., 2009; Venugopal et al., 2020). Interestingly, cells that exhibit aberrant cell cycle control (e.g., tumor cells) do not generally possess primary cilia at any phase of the cell cycle (Wheatley, 1995). Taken together, our data suggest that the primary cilium is capable of acting as a key signaling node for perceiving shear stress on endothelial cells and mediating the stimulus of laminar flow on KLF2 expression, at least in part, through activation of SSTR3.

GPR101 was also found to modulate KLF2 expression in endothelial cells. Selective knockdown of GPR101 robustly stimulated KLF2 expression and was associated with an anti-inflammatory gene expression profile. These data suggest that GPR101 in endothelial cells also exerts a tonic negative effect on KLF2 expression. This was supported by a reduction in TNFα-induced monocyte adhesion to endothelial cells in culture. Interestingly, we did not observe an impact of GPR101 KD on flow dependent KLF2 expression suggesting that despite modulating KLF2 expression, GPR101 does not seem to be responsible for regulation of KLF2 expression induced by laminar shear. Recent published data has suggested that the proresolvin, RvD5_n-3DPA_, is a high affinity (pM) ligand for GPR101 and that GPR101 mediates the pro-resolution effects of this resolvin (Flak et al., 2020). The authors propose that activation of GPR101 results in an anti-inflammatory effect on macrophages and induces phagocytosis through a cholera toxin-sensitive pathway. We have observed that forskolin can stimulate KLF2 expression in endothelial cells. In contrast to the effects of forskolin, KD of GPR101 in endothelial cells was clearly stimulatory to the anti-inflammatory KLF2 transcriptional program in endothelial cells. However, we did not observe an effect of, RvD5_n-3DPA_ on KLF2 expression in HUVECs (Supplementary Figure S6). Taken together, these data suggest that GPR101 may mediate pro-inflammatory pathways in immune and endothelial cells. It remains unclear if this is through a Gs-mediated signaling event since there are no reports of ligand-directed Gs activation or elevation in cAMP via GPR101.

We have also reported here that LGR4 regulates KLF2 expression independent of laminar shear. LGR4 is related to a leucine-rich repeat sub-family of GPCRs that also includes receptors to the glycoprotein hormones (LH, FSH and TSH) and relaxin (RXFP1) (Hsu et al., 2000). LGR4 immunoreactivity was present in the endothelium of human tissues and cultures of endothelial cells. KD of LGR4 reduced KLF2 expression in static cultures of endothelial cells but did not affect flow dependent KLF2 expression. Over-expression of LGR4 in HUVECs significantly elevated KLF2 and KLF2 target genes (eNOS and TM). As a proof of concept to developing drug discovery targets from the identified targets, we performed a high-throughput screen of the BMS library to identify compounds capable of activating LGR4. We chose to utilize a proximal signaling end point (β-arrestin recruitment) since we had not observed increased cAMP in the presence of RSPO1 in HUVECs or in EA-hy926 cells over-expressing LGR4. The rationale for this choice lay in the fact that the majority of GPCRs are capable of recruiting *ß*-arrestin and therefore this end point represented a more G protein agnostic approach to identifying novel small molecule ligands. We identified several chemotypes that were capable of stimulating LGR4-dependent *ß*-arrestin recruitment in our screen. Most notably, the two compounds identified in HTS and used in validation of the strategy (Compound 1 and Compound 2) both share a biaryl, tetrazole core structure that is shared with well-known clinical therapeutics targeting the angiotensin receptor 1 (AT1R). Interestingly, here we have observed that compounds with this core structure can act as agonists of LGR4 whereas at AT1R, these compounds are known to be competitive antagonists (Sica, 2001). These compounds were also capable of dose-dependently stimulating a KLF2 promoter luciferase reporter gene that was eliminated in the presence of LGR4 shRNA. The impact of Compound 1 on endothelial function was demonstrated through concentration-dependent improvements in endothelial barrier function, monocyte adhesion and cytokine release (MCP-1), thus reducing to practice the goal of identifying GPCRs capable of modulating KLF2 expression and thereby improving endothelial function. Potencies of Compound 1 and Compound 2 in endothelial functional assays were consistent with their respective potencies for recruiting *ß*-arrestin. Although weak, these compounds potentially represent the first known reported synthetic ligands for LGR4. Perhaps more importantly, they validate our approach to phenotypically identifying pathway modulating GPCRs that can ultimately be screened for identifying small molecule compounds capable of starting drug discovery activities. Further work is required to improve and understand compound affinity and selectivity. These compounds did not increase cAMP production in endothelial cells or HMEC cells (data not shown). Likewise, these compounds did not stimulate the beta-catenin signaling pathway as has been shown for RSPOs (Carmon et al., 2011; Kang et al., 2016) suggesting they are signaling through LGR4 in a unique manner.

Although the potencies of these compounds to stimulate the KLF2 transcriptional program were low to double-digit micromolar, these compounds did demonstrate the requirement for LGR4 expression for these effects. Knockdown of LGR4 significantly reduced compound efficacy on KLF2 expression and KLF2 promoter-luciferase activity. Additionally, these compounds represent reasonable potential starting points for optimization efforts toward identifying therapeutically relevant candidates. However, it should be noted that, although we were able to identify receptors and compounds capable of modulating KLF2 expression *in vitro*, in these experiments we did not demonstrate effects on KLF2 expression or endothelial function *in vivo*. It remains to be determined if synthetic agonists/antagonists to these receptors, and optimized for administration to animals, can lead to improved endothelial function within the context of a suitable animal model.

In summary, we have identified a population of GPCRs capable of affecting the KLF2 transcriptional program within human endothelial cells. Some of these receptors were demonstrated to have potential as transducers of mechanical shear force consistent with the key role of KLF2 in translating mechanical forces to a beneficial transcriptional program within these cells. Lastly, we have developed a process for the development of drug discovery efforts around LGR4 as an exemplar of how pathway-specific phenotypic screening can successfully lead to identification of putative chemical matter for this target class.

## Data Availability

The original contributions presented in the study are included in the article/Supplementary Material, further inquiries can be directed to the corresponding author.
