# Supplementary material for: Identification and validation of G protein-coupled receptors modulating flow-dependent signaling pathways in vascular endothelial cells
Source: Front Mol Biosci. 2023 Jun 8;10:1198079. doi: 10.3389/fmolb.2023.1198079 (PMC10285409; doi:10.3389/fmolb.2023.1198079)

A.

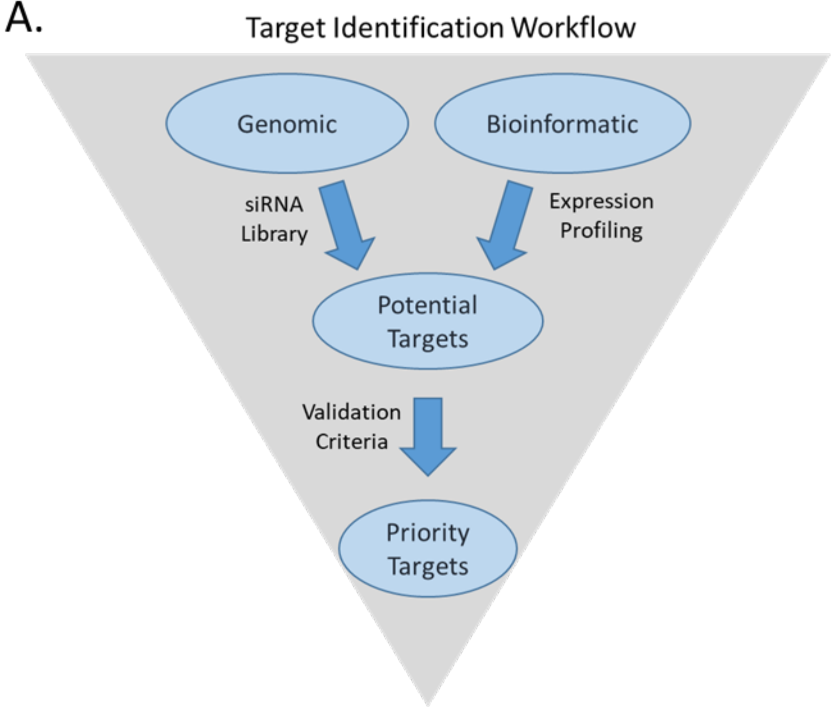

B.

Average Expression	Frequency of Appearance	Gene Name
2179.7	3	Caveolin 1, caveolae protein, 22kDa
773.7	3	folate receptor 1 (adult)
352.3	3	Coagulation factor II (Thrombin) receptor-like 1
297.3	3	frizzled homolog 6 (Drosophila)
287.0	3	PTK2 protein tyrosine kinase 2
279.3	3	G protein-coupled receptor 126
266.3	3	EGF, latrophilin and seven transmembrane domain containing 1
254.3	3	prostaglandin E receptor 4 (subtype EP4)
228.7	3	Coagulation factor II (Thrombin) receptor
139.3	3	met proto-oncogene (hepatocyte growth factor receptor)
138.2	3	G protein-coupled receptor 89A
136.0	3	Neuroepithelial cell transforming gene 1
130.4	3	Endothelial differentiation, sphingolipid G-protein-coupled receptor, 1
127.0	3	Calcitonin receptor-like
115.0	3	Histamine receptor H1
107.0	3	Endothelial differentiation, lysophosphatidic acid G-protein-coupled receptor, 2
87.8	3	chemokine (C-X-C motif) receptor 7
84.2	3	EPH receptor B2
79.8	3	opsin 3 (encephalopsin, panopsin)
72.9	3	Aryl hydrocarbon receptor
71.9	3	G protein-coupled receptor 116
65.9	3	Dopamine receptor D2
61.7	3	Glutamate receptor, metabotropic 6
59.9	3	Purinergic receptor P2Y, G-protein coupled, 5
58.3	3	Latrophilin 2
47.5	3	cadherin, EGF LAG seven-pass G-type receptor 2 (flamingo homolog, Drosoph
46.5	3	G protein-coupled receptor 27
44.7	3	G protein-coupled receptor 137B
40.6	3	Dopamine receptor D5
39.2	3	Pyrimidinergic receptor P2Y, G-protein coupled, 4
35.8	3	G protein-coupled receptor 161
27.5	3	leucine-rich repeat-containing G protein-coupled receptor 4
26.6	3	frizzled homolog 5 (Drosophila)
25.2	3	Adenosine A1 receptor
22.1	3	Chemokine (C-C motif) receptor 9
274.3	2	G protein-coupled receptor, family C, group 5, member A
175.0	2	Adenosine A2b receptor
81.3	2	G protein-coupled receptor, family C, group 5, member B
65.9	2	Oxytocin receptor
64.9	2	frizzled homolog 2 (Drosophila)
55.4	2	Latrophilin 1
54.2	2	Mitogen-activated protein kinase 3
52.5	2	Adrenergic, beta-2-, receptor, surface
52.2	2	cannabinoid receptor 2 (macrophage)
49.4	2	frizzled homolog 7 (Drosophila)
48.1	2	G protein-coupled receptor 137
41.5	2	Endothelin receptor type A
40.6	2	G protein-coupled receptor 176
31.9	2	trace amine associated receptor 2
25.9	2	Prostaglandin E receptor 2 (Subtype EP2), 53kDa
23.3	2	5-hydroxytryptamine (Serotonin) receptor 2A
19.7	2	Angiotensin II receptor, type 1
16.4	2	Tachykinin receptor 1
15.1	2	cadherin, EGF LAG seven-pass G-type receptor 1 (flamingo homolog, Drosoph
11.3	2	adrenergic, alpha-1A-, receptor
10.1	2	5-hydroxytryptamine (Serotonin) receptor 2C
7.1	2	G protein-coupled receptor 1
225.9	1	frizzled homolog 4 (Drosophila)
200.5	1	Chemokine (C-X-C motif) receptor 4
83.4	1	Thromboxane A2 receptor
73.9	1	G protein-coupled receptor 56
61.6	1	gamma-aminobutyric acid (GABA) B receptor, 2
61.2	1	G protein-coupled receptor 124
45.1	1	Neuropeptide Y receptor Y5
38.3	1	G protein-coupled receptor 21
33.0	1	retinal pigment epithelium-derived rhodopsin homolog
30.1	1	Gonadotropin-releasing hormone receptor
29.8	1	G protein-coupled receptor 35
29.4	1	Neuropeptide Y receptor Y2
24.3	1	CD97 molecule
23.5	1	Histamine receptor H4
21.6	1	Peroxisome proliferator-activated receptor gamma
20.0	1	Endothelin receptor type B
18.5	1	G protein-coupled receptor 68
17.8	1	G protein-coupled receptor 39
16.6	1	cannabinoid receptor 1 (brain)
14.2	1	Brain-specific angiogenesis inhibitor 3
13.9	1	Glutamate receptor, metabotropic 8
5.3	1	Cysteinyl leukotriene receptor 1
4.9	1	Chemokine (C-C motif) receptor 6

Supplemental Figure S1. Target Identification Strategy and Targets Derived from Bioinformatics

Supplemental Figure S2. Validation of 96 well Orbital Flow Assay

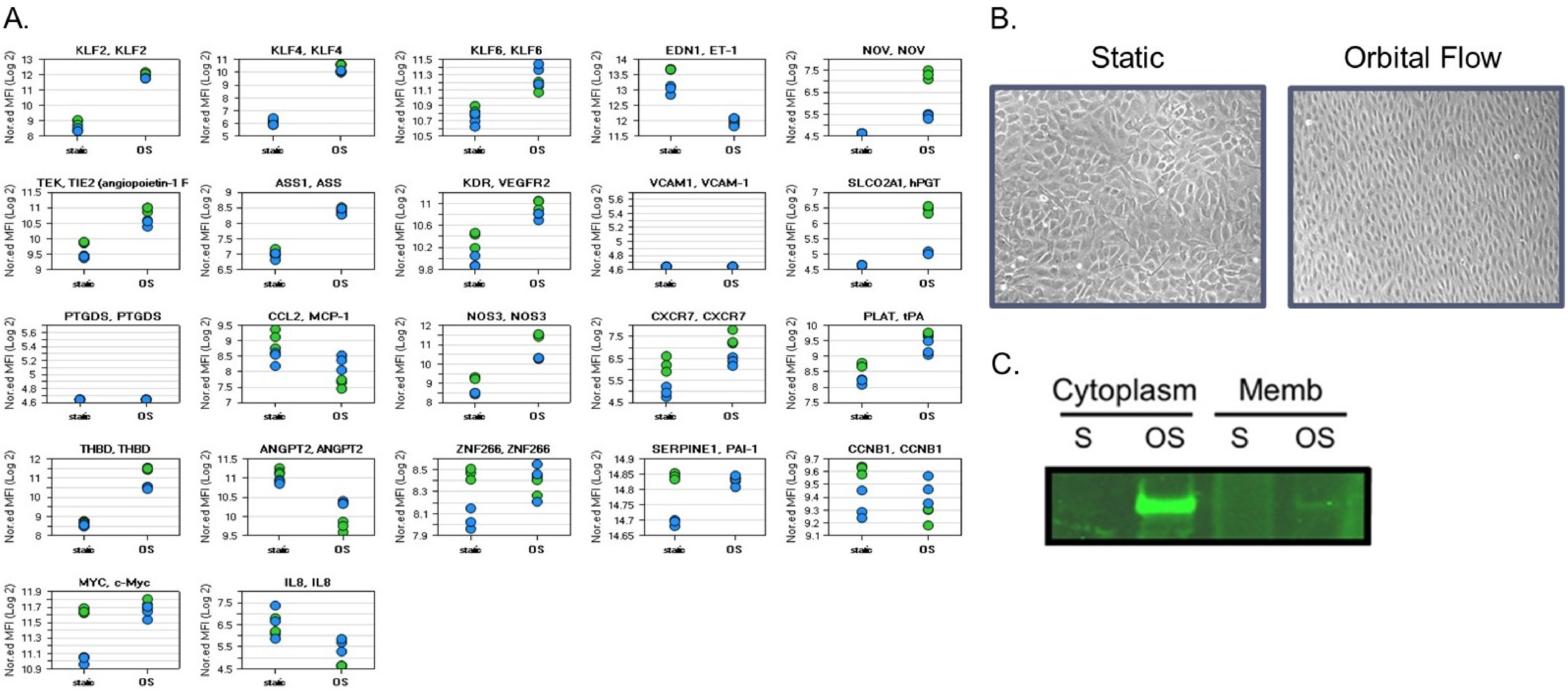

Supplemental Figure S3. Time-course of Orbital Flow Effects on LGR4 and KLF2 Expression in HUVECs

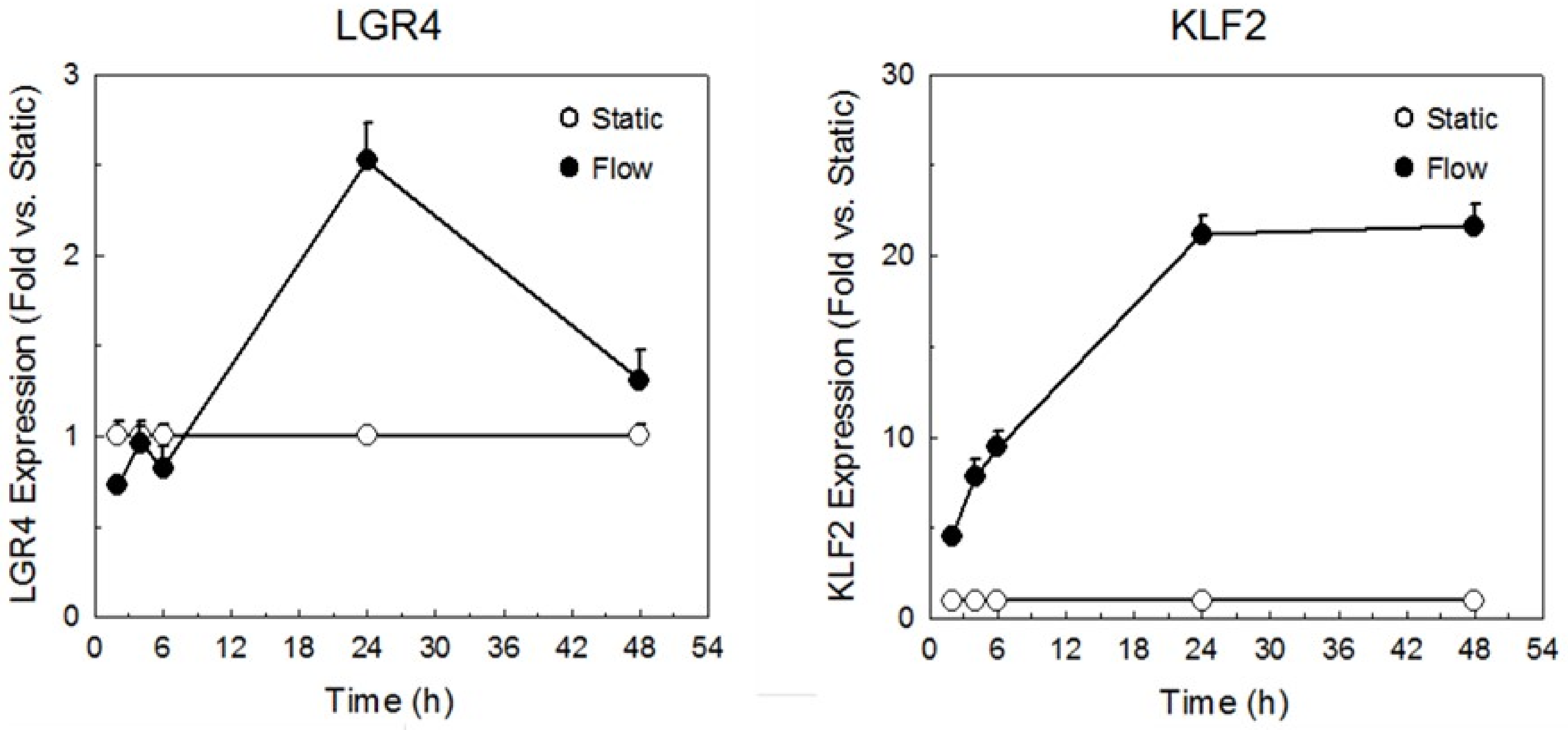

Supplemental Figure S4. Overexpression of LGR4 in EA-hy926 cells stimulates KLF2 Promoter Activity

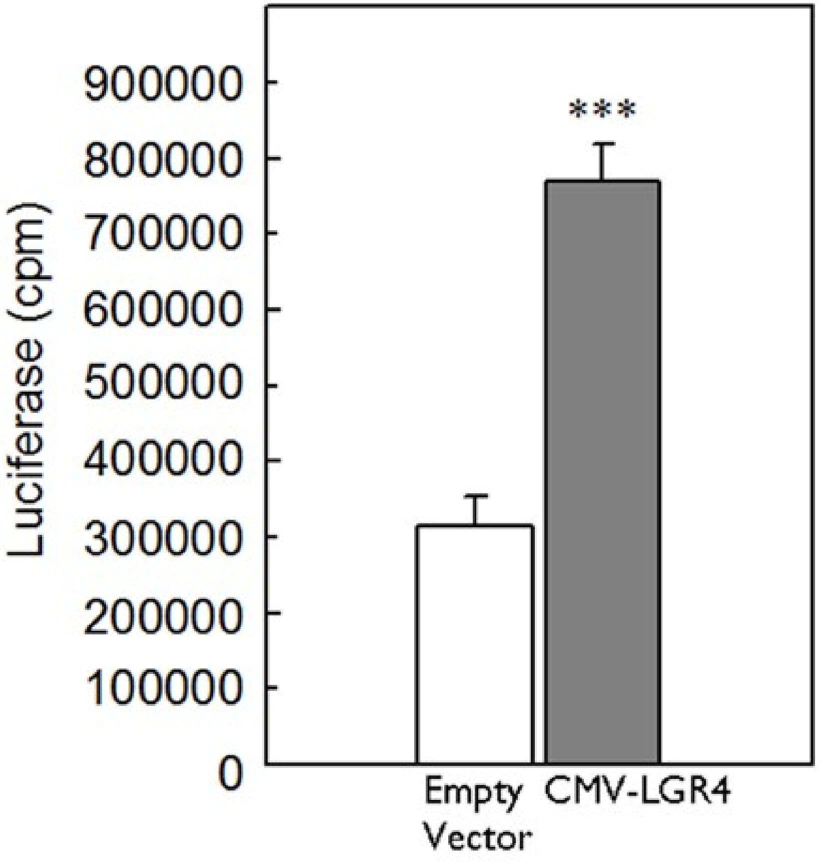

Supplemental Figure S5. Structures of Putative LGR4 Agonists Compound 1 and Compound 2

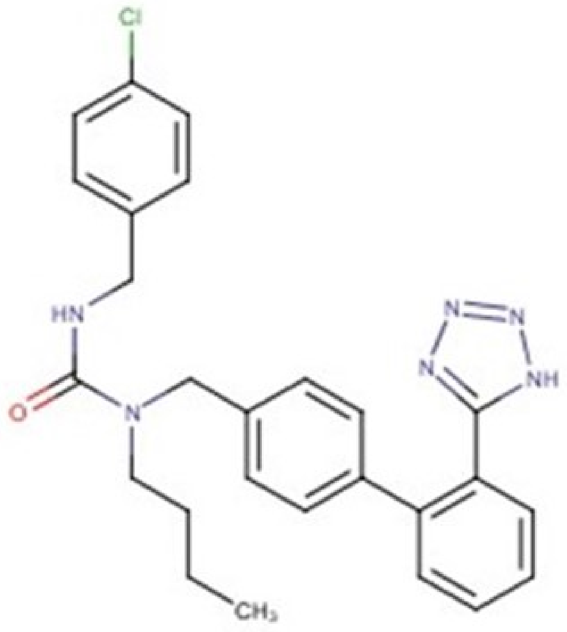

Compound 1

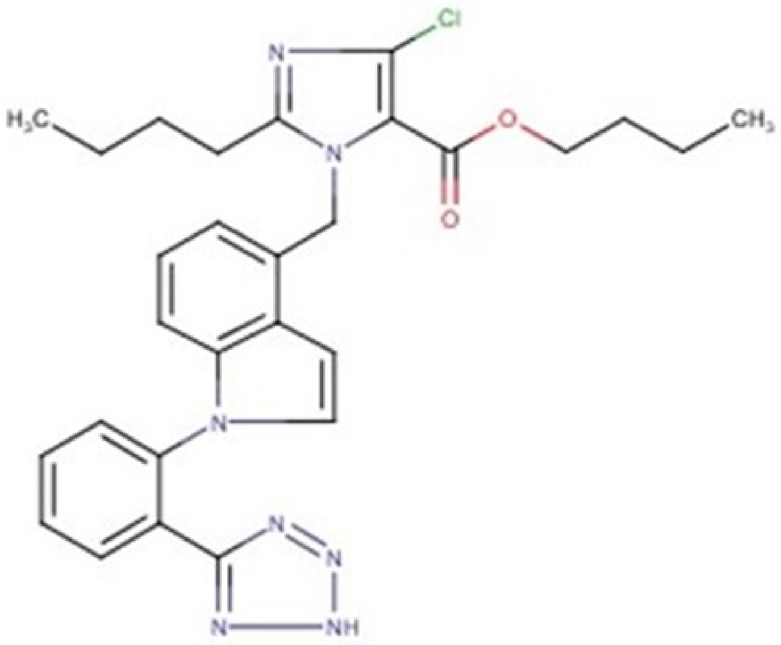

Compound 2

Supplemental Figure S6. Effect of Resolvin, $RvD5_{n-3DPA}$, on KLF2 Expression in HUVEC cells

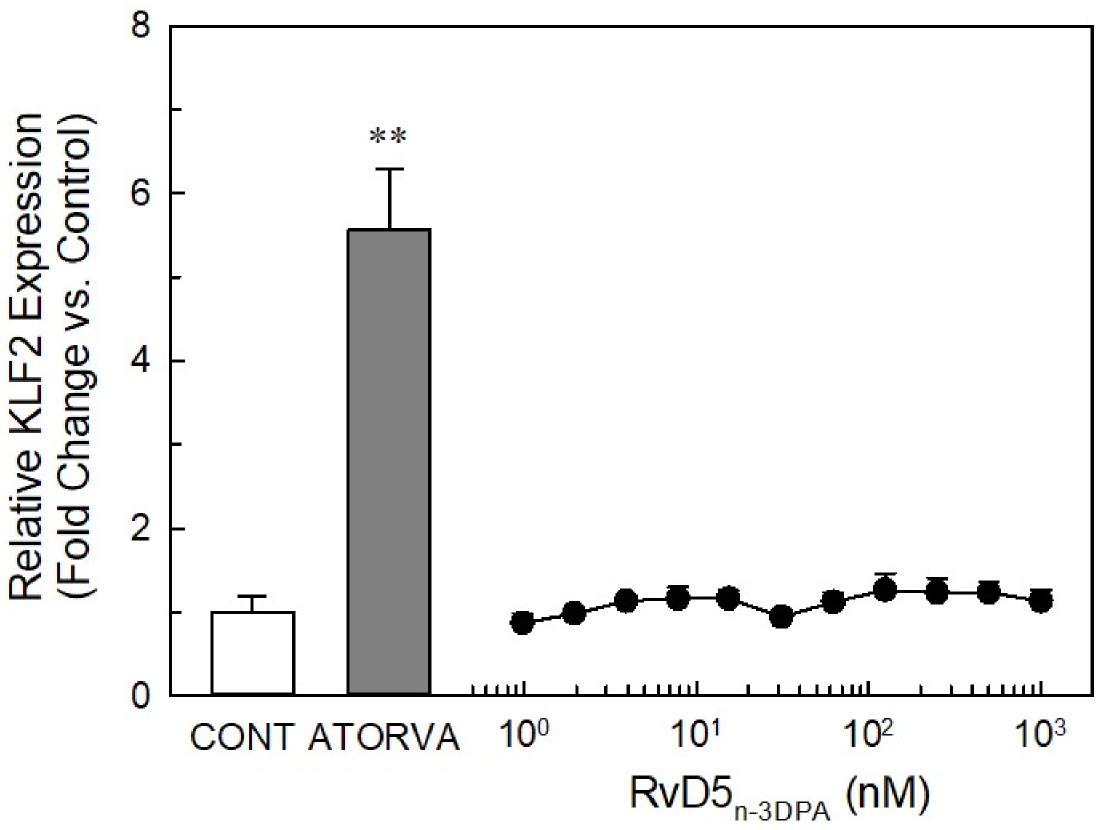

Supplement: Supplementary file 1 [file Presentation1.pdf]
