# Supplementary material for: Identification and validation of G protein-coupled receptors modulating flow-dependent signaling pathways in vascular endothelial cells
Source: Front Mol Biosci. 2023 Jun 8;10:1198079. doi: 10.3389/fmolb.2023.1198079 (PMC10285409; doi:10.3389/fmolb.2023.1198079)
Supplement: Supplementary file 2 [file Presentation2.PPTX]

## Slide 1
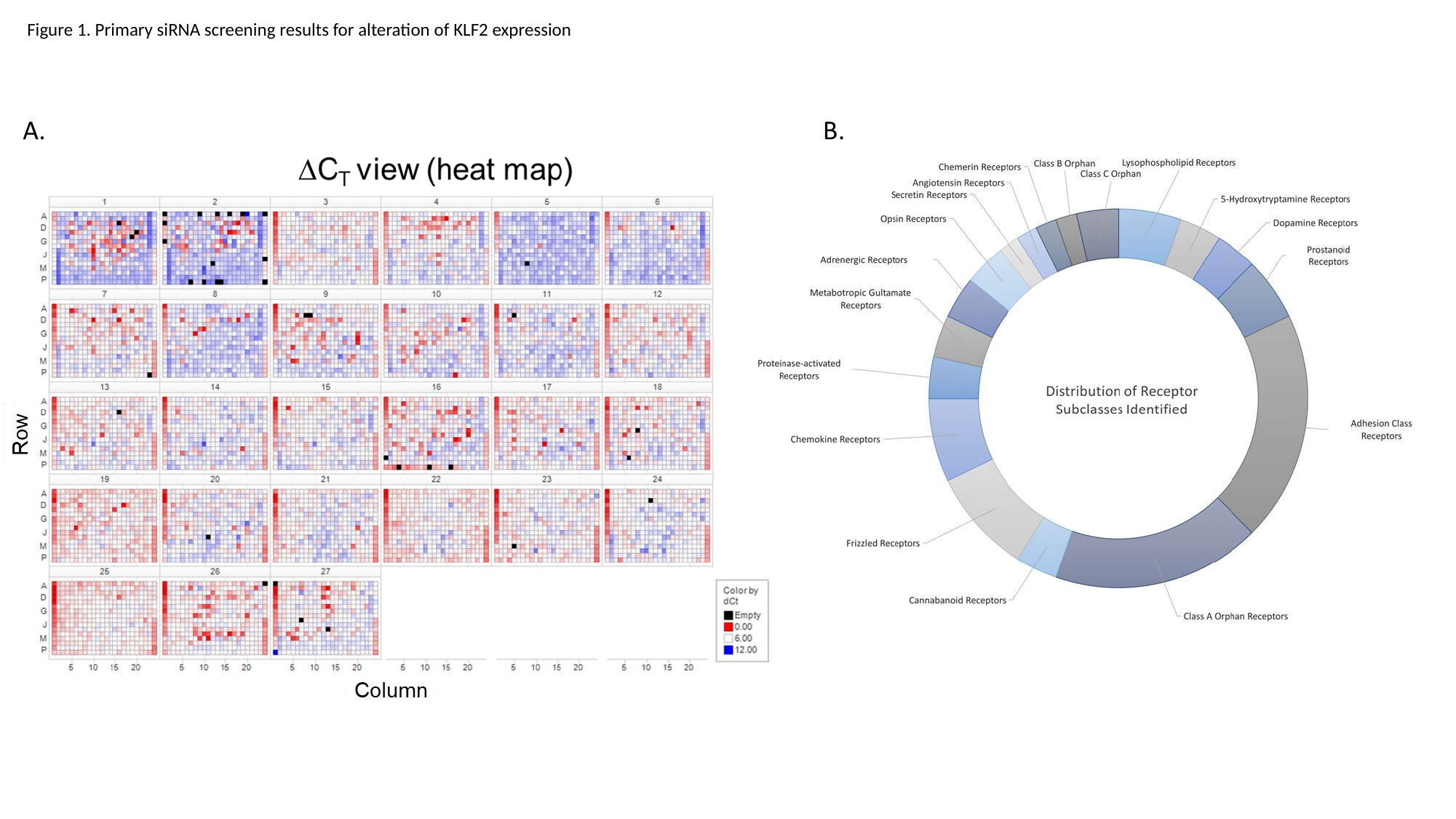

Figure 1. Primary siRNA screening results for alteration of KLF2 expression

## Slide 2
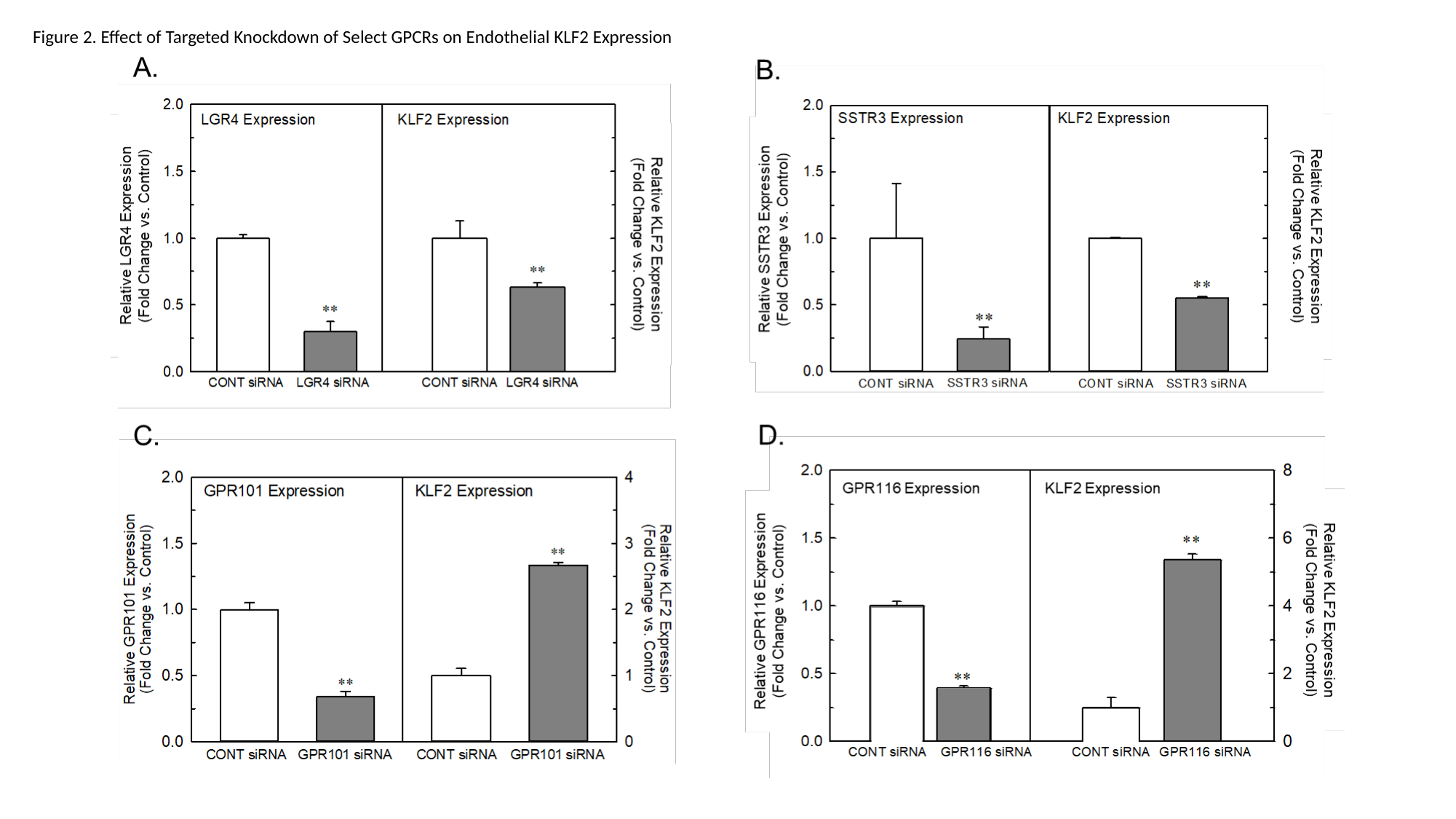

Figure 2. Effect of Targeted Knockdown of Select GPCRs on Endothelial KLF2 Expression

## Slide 3
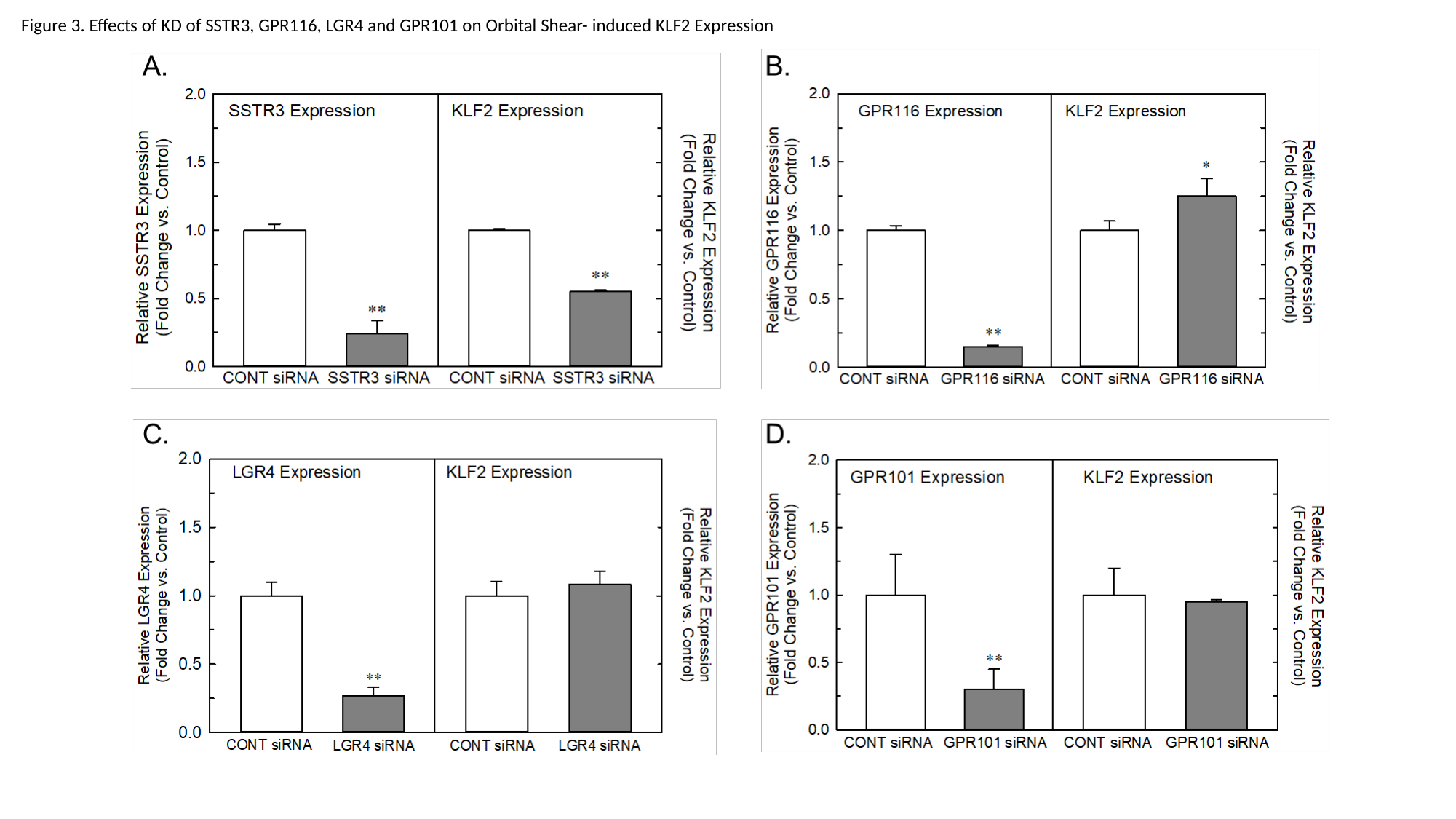

Figure 3. Effects of KD of SSTR3, GPR116, LGR4 and GPR101 on Orbital Shear- induced KLF2 Expression

## Slide 4
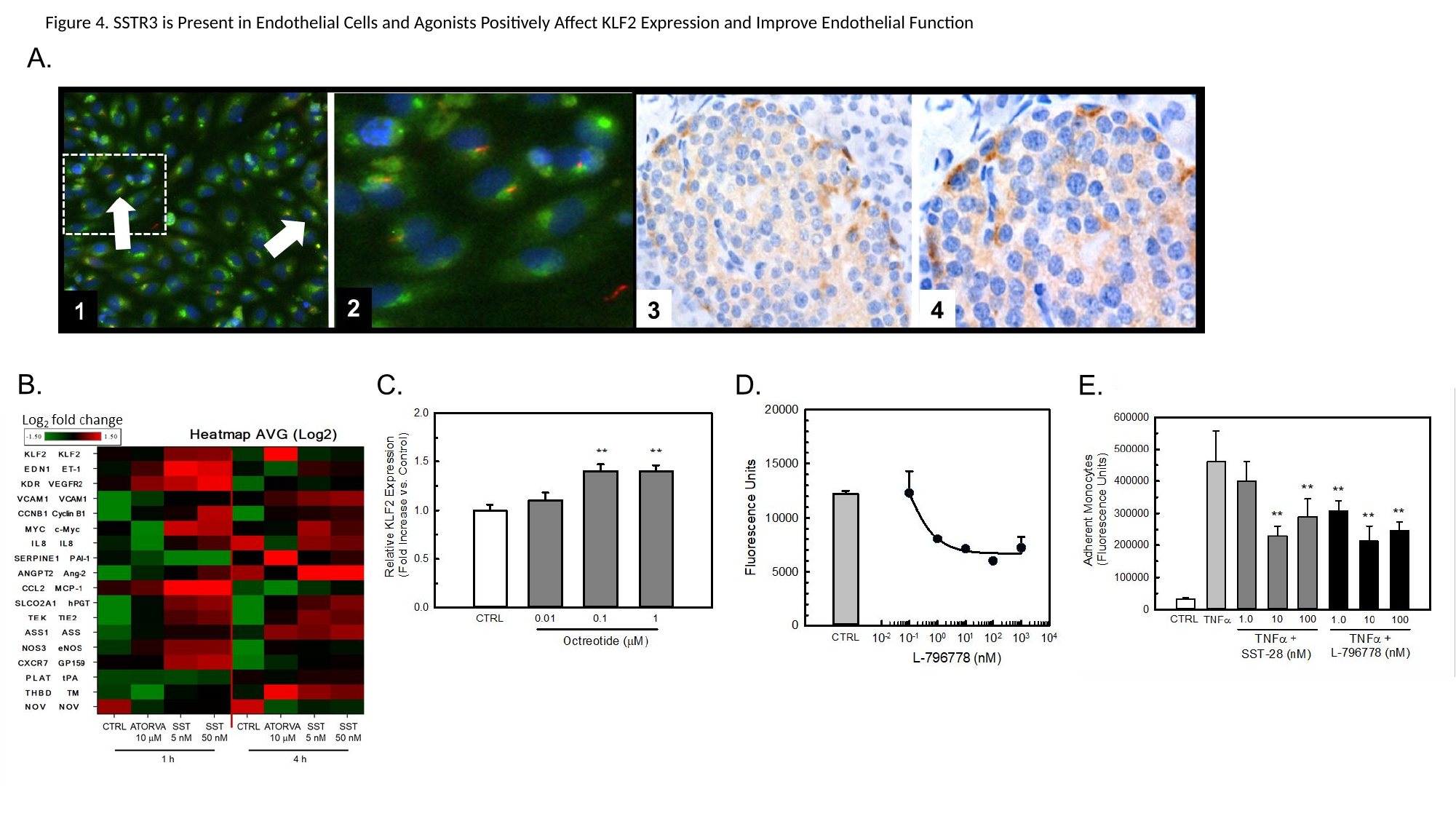

Figure 4. SSTR3 is Present in Endothelial Cells and Agonists Positively Affect KLF2 Expression and Improve Endothelial Function

## Slide 5
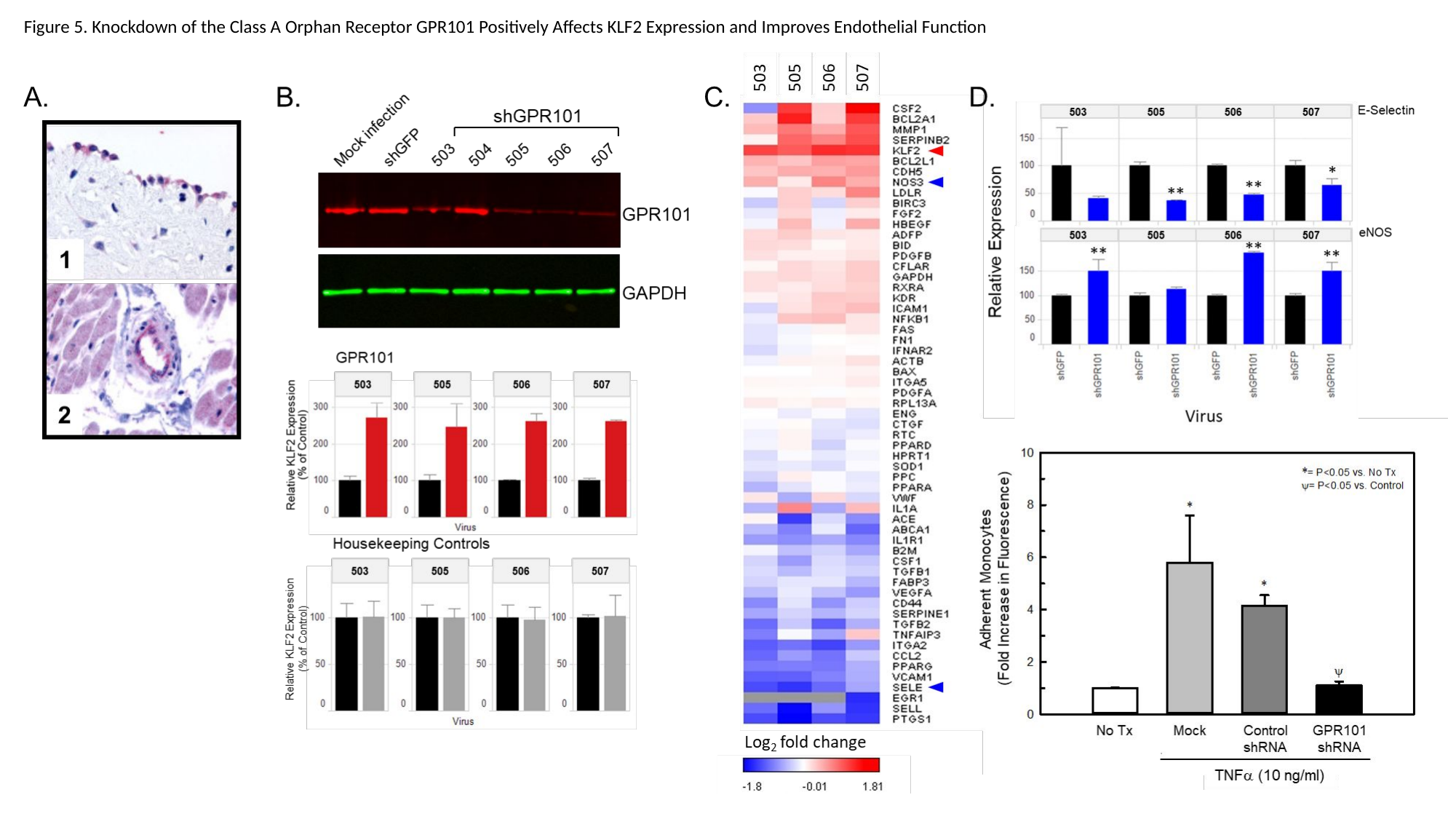

Figure 5. Knockdown of the Class A Orphan Receptor GPR101 Positively Affects KLF2 Expression and Improves Endothelial Function

## Slide 6
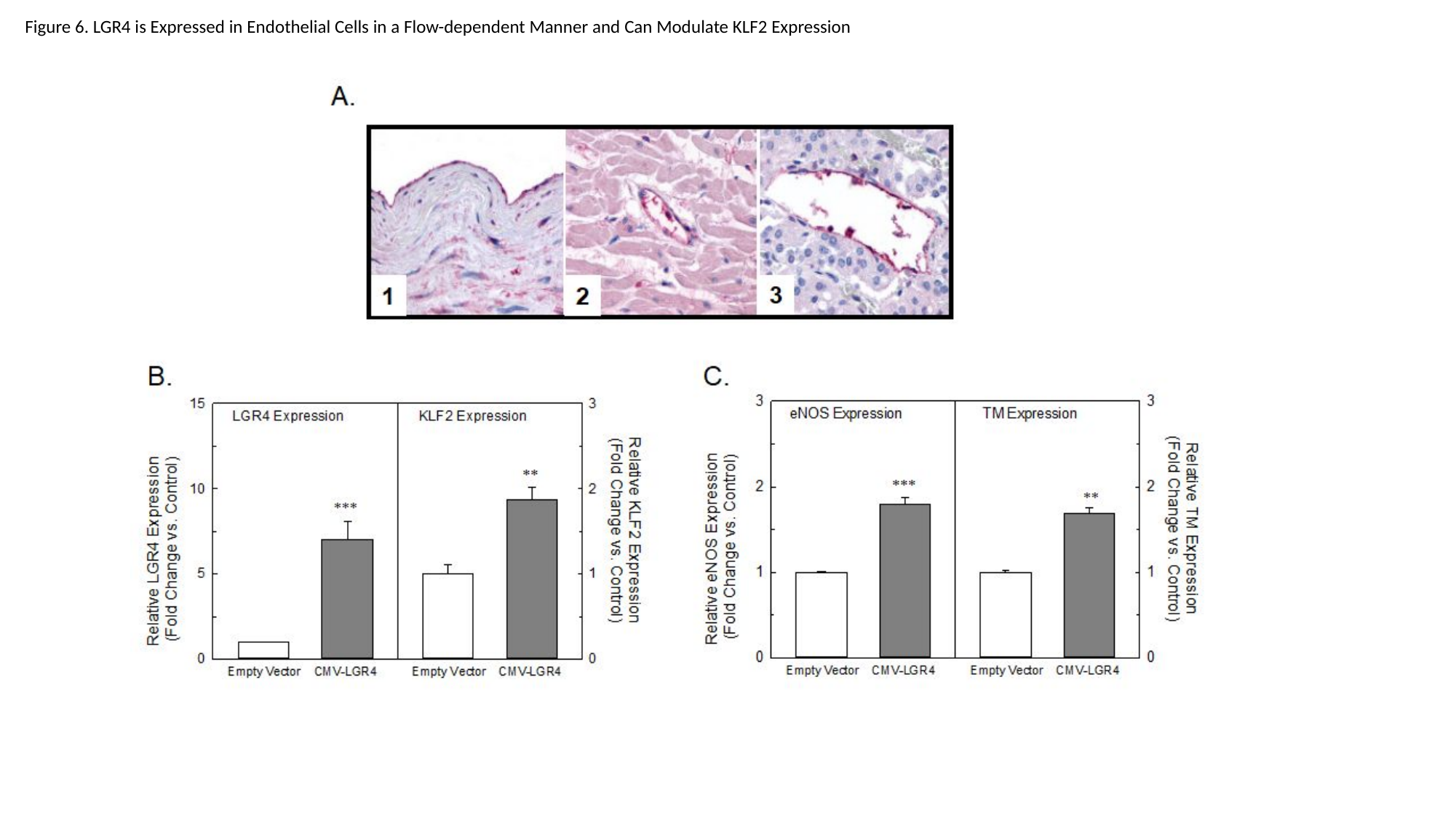

Figure 6. LGR4 is Expressed in Endothelial Cells in a Flow-dependent Manner and Can Modulate KLF2 Expression

## Slide 7
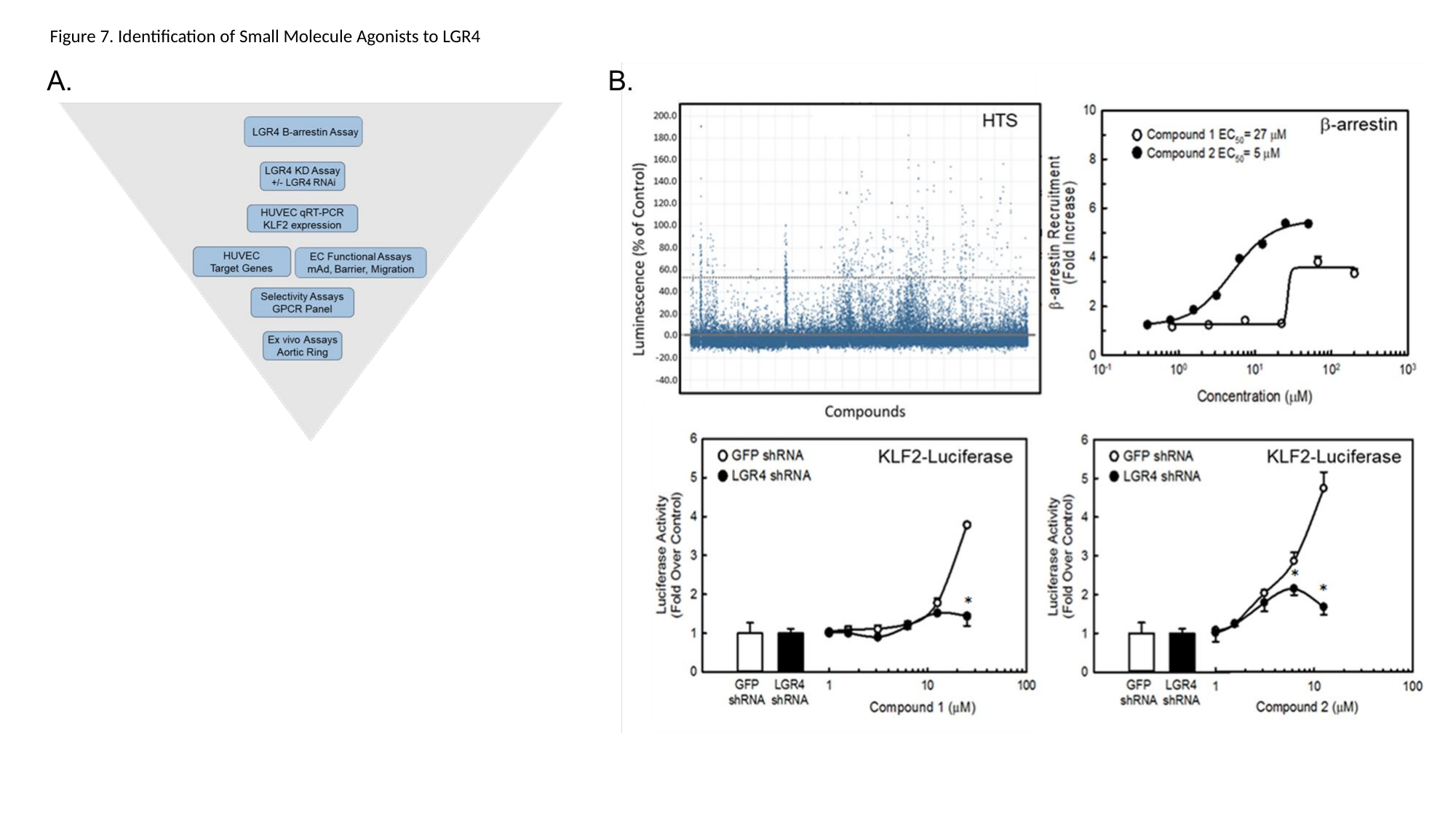

Figure 7. Identification of Small Molecule Agonists to LGR4

## Slide 8
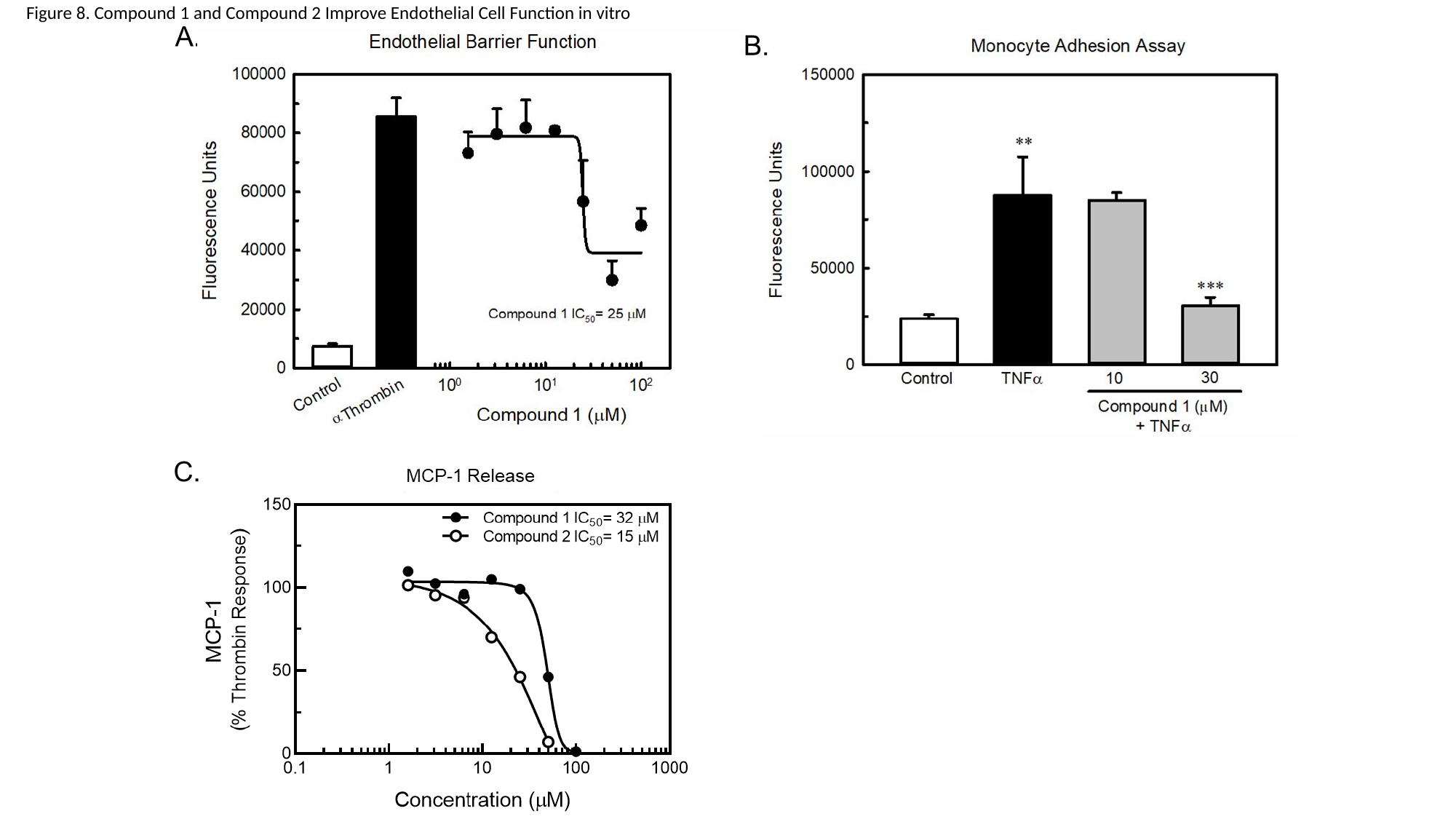

Figure 8. Compound 1 and Compound 2 Improve Endothelial Cell Function in vitro

## Slide 9
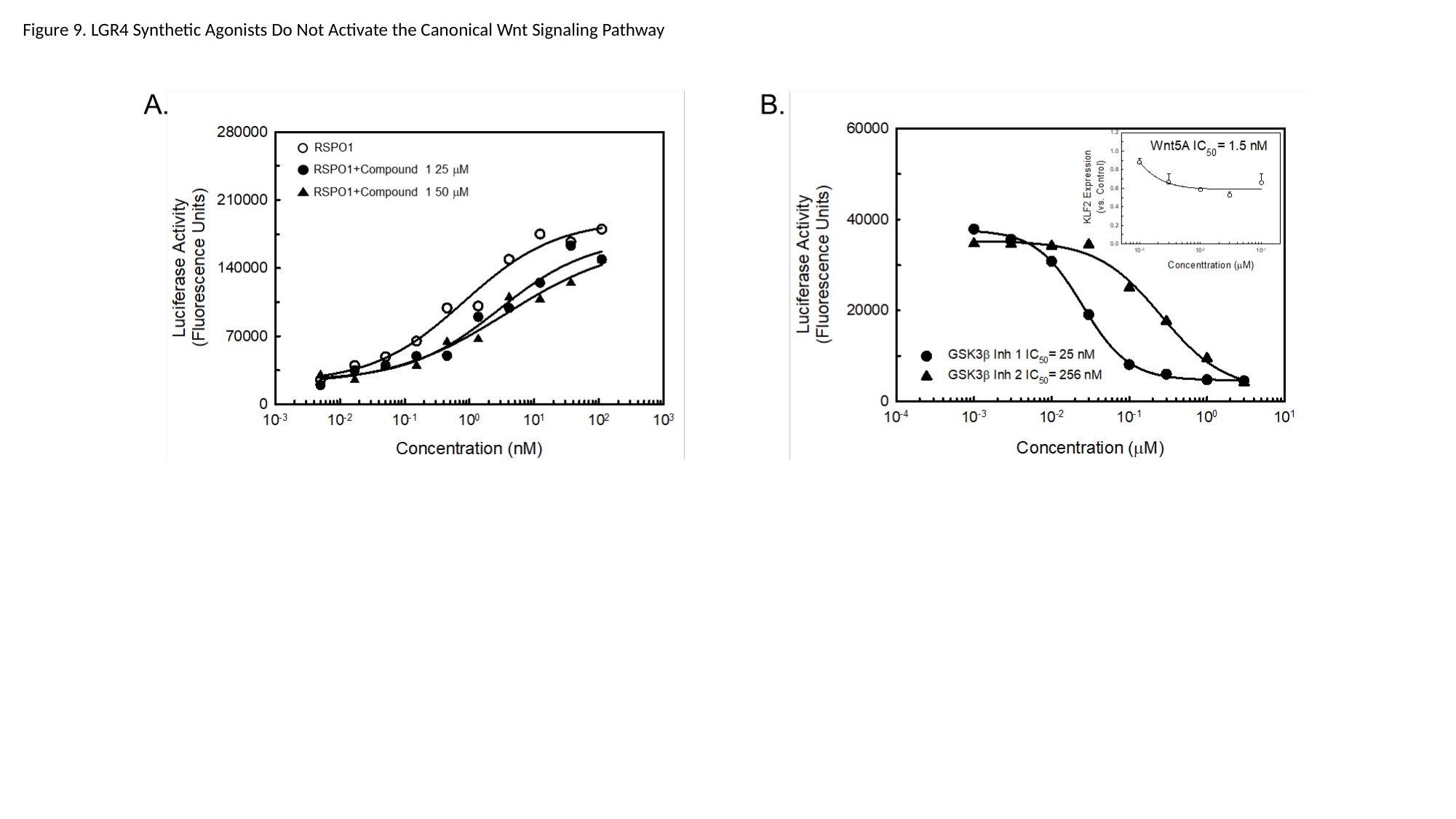

Figure 9. LGR4 Synthetic Agonists Do Not Activate the Canonical Wnt Signaling Pathway

## Slide 10
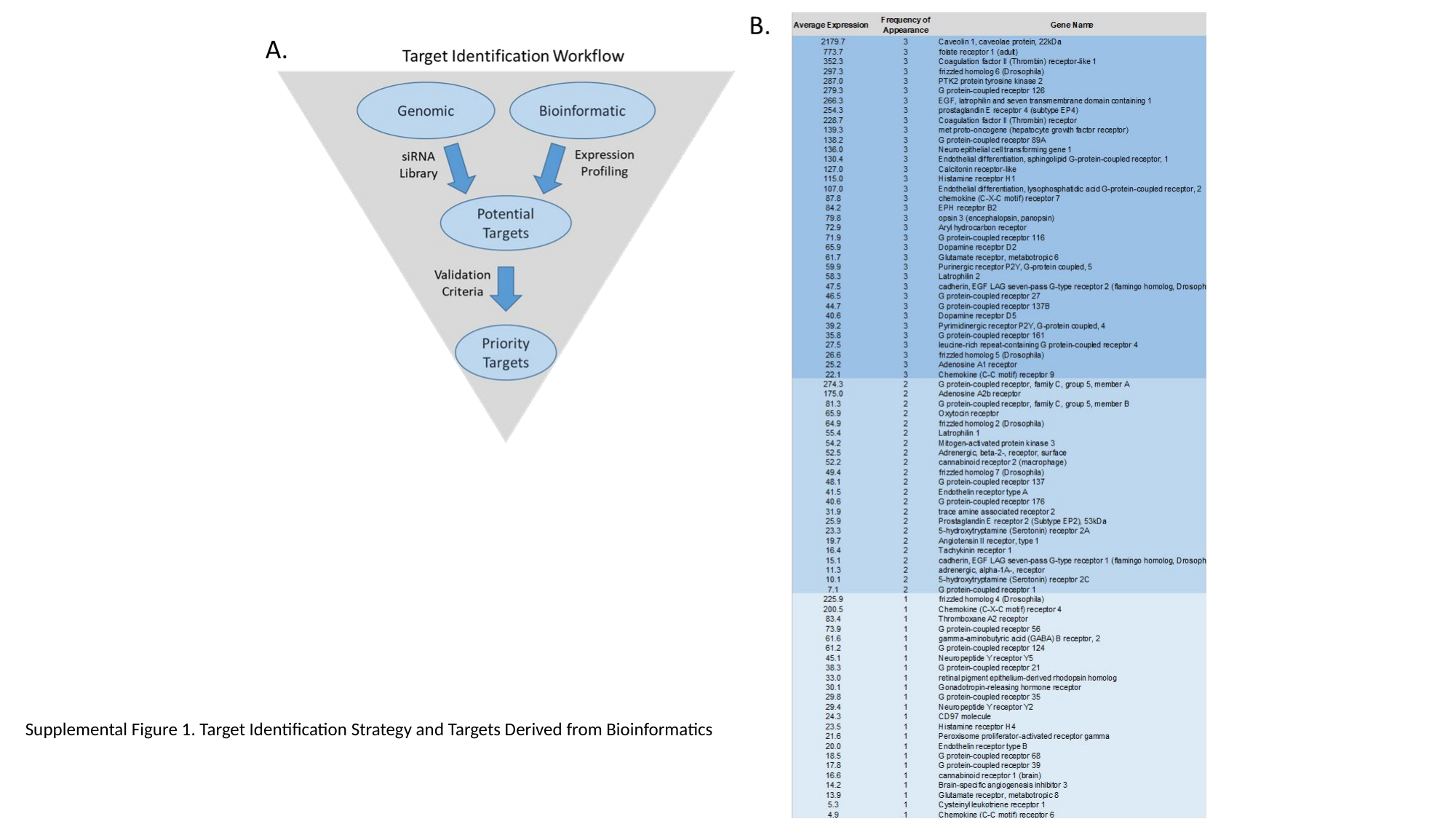

Supplemental Figure 1. Target Identification Strategy and Targets Derived from Bioinformatics

## Slide 11
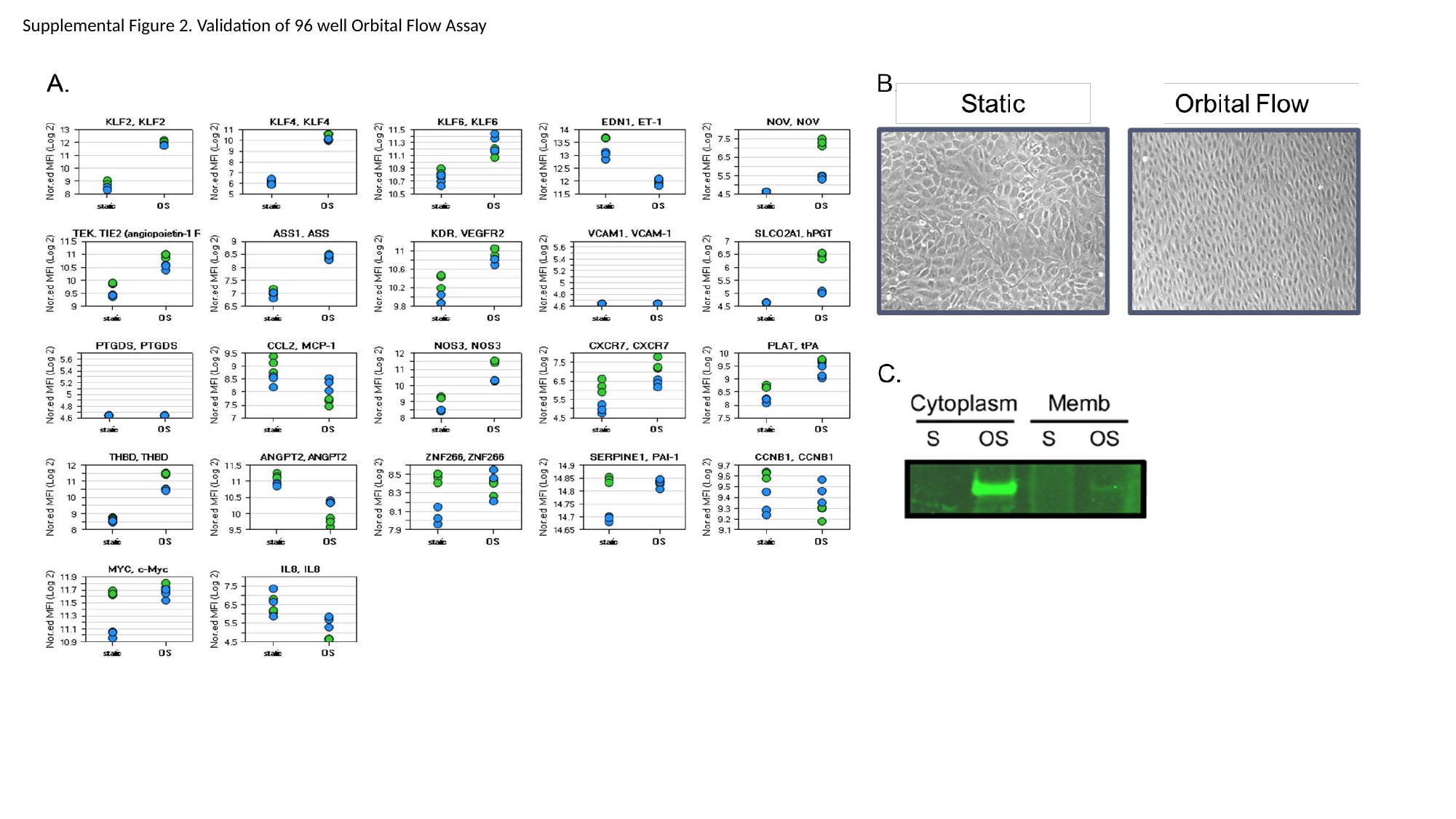

Supplemental Figure 2. Validation of 96 well Orbital Flow Assay

## Slide 12
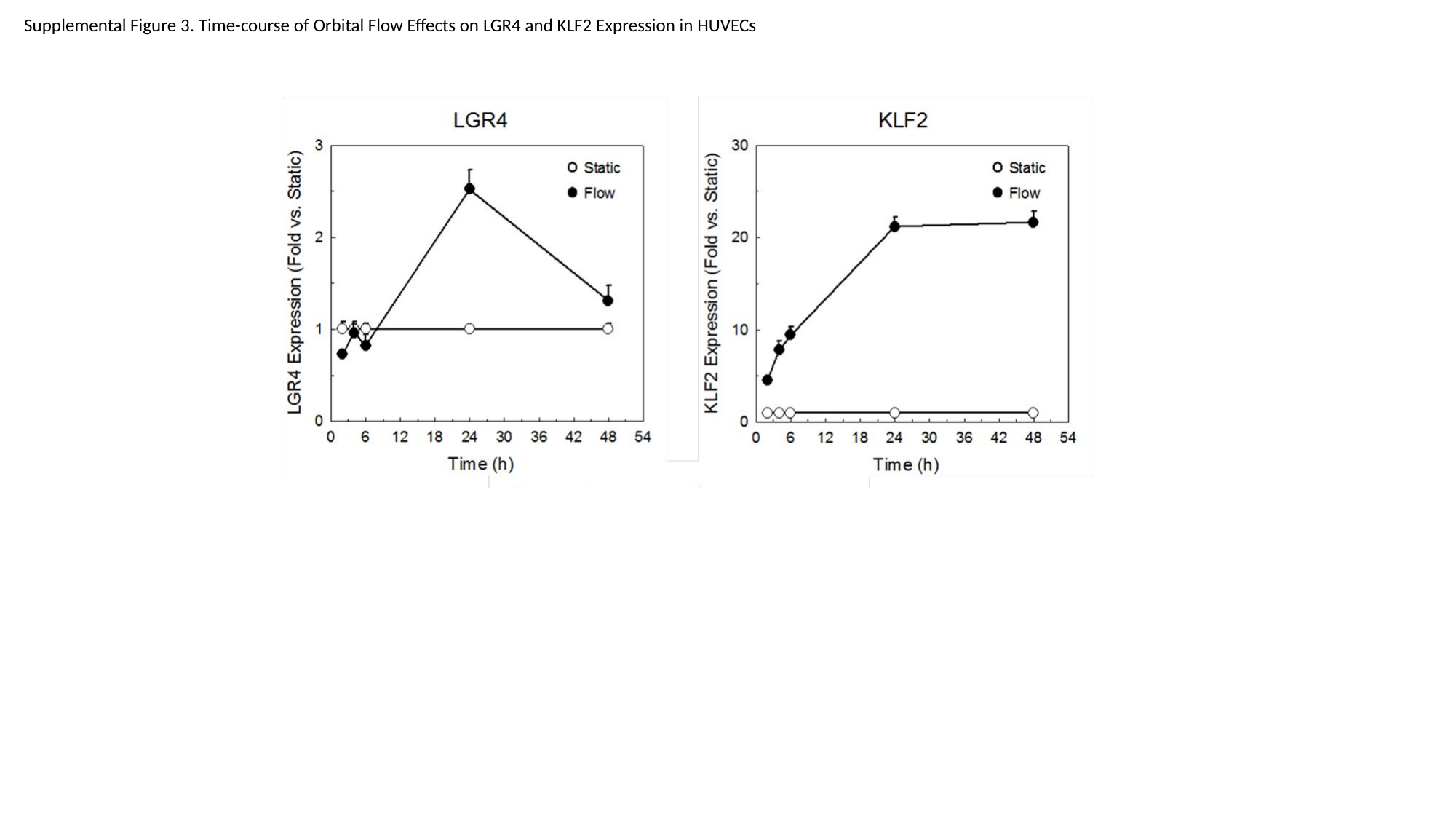

Supplemental Figure 3. Time-course of Orbital Flow Effects on LGR4 and KLF2 Expression in HUVECs

## Slide 13
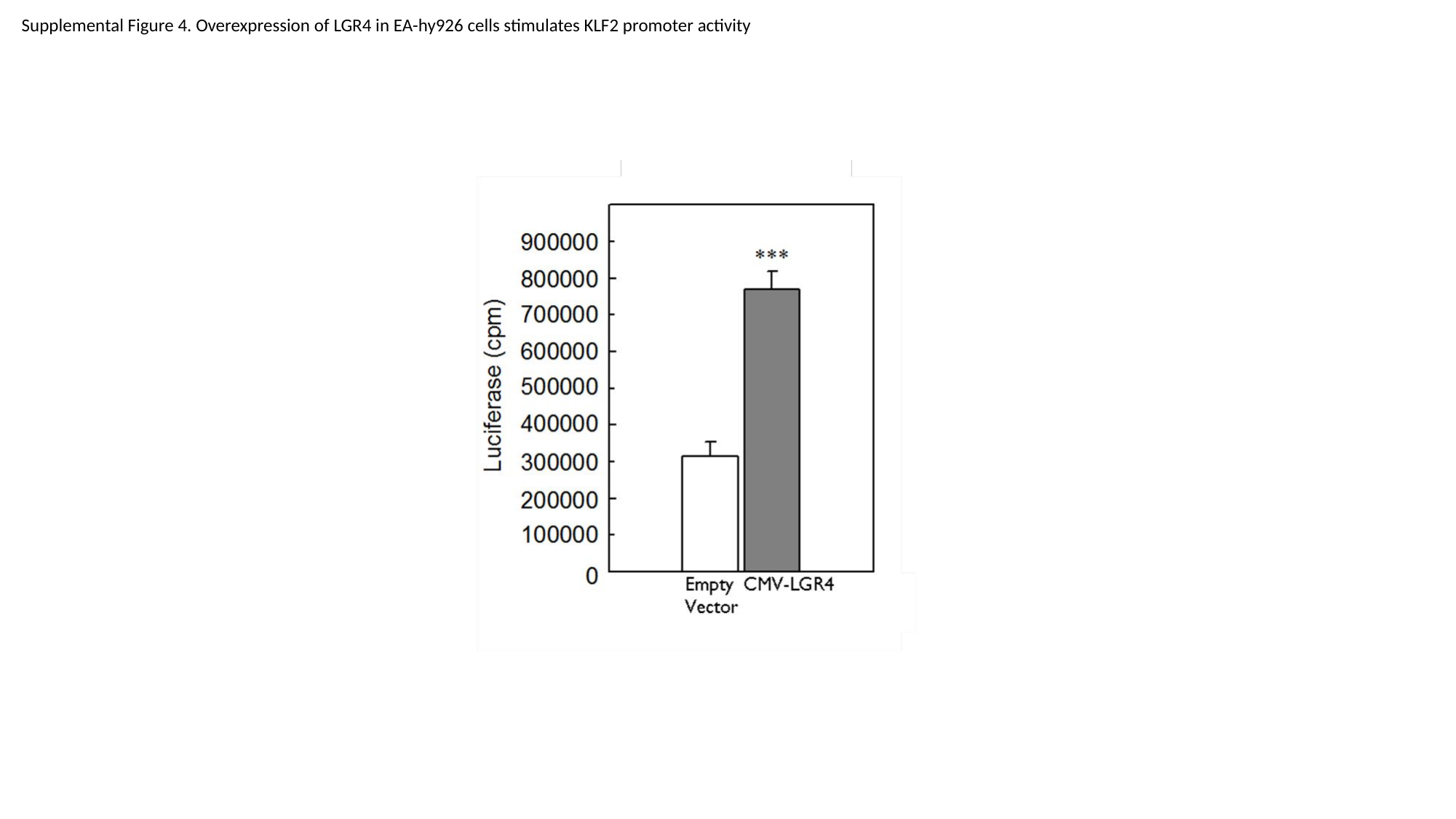

Supplemental Figure 4. Overexpression of LGR4 in EA-hy926 cells stimulates KLF2 promoter activity

## Slide 14
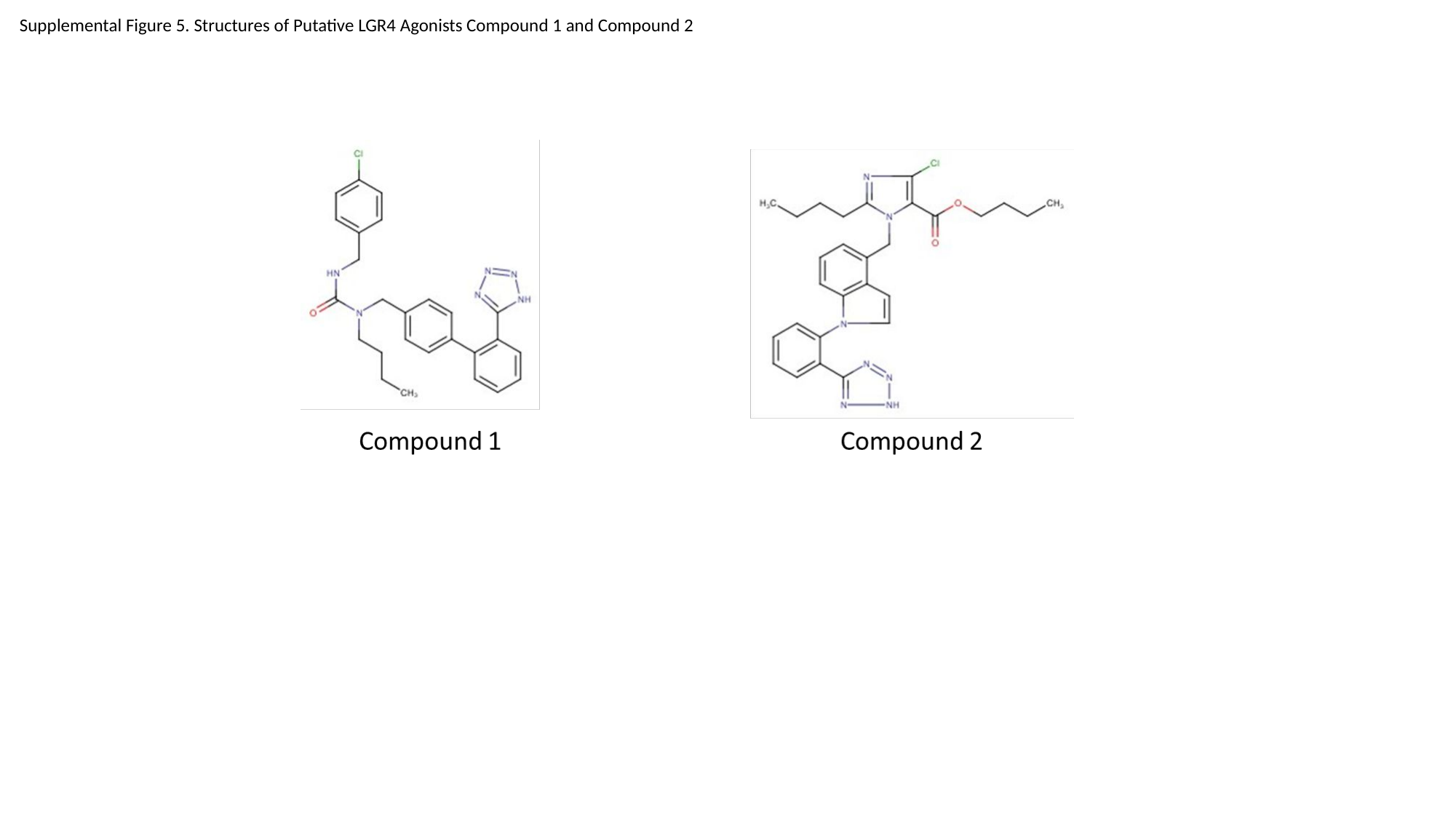

Supplemental Figure 5. Structures of Putative LGR4 Agonists Compound 1 and Compound 2

## Slide 15
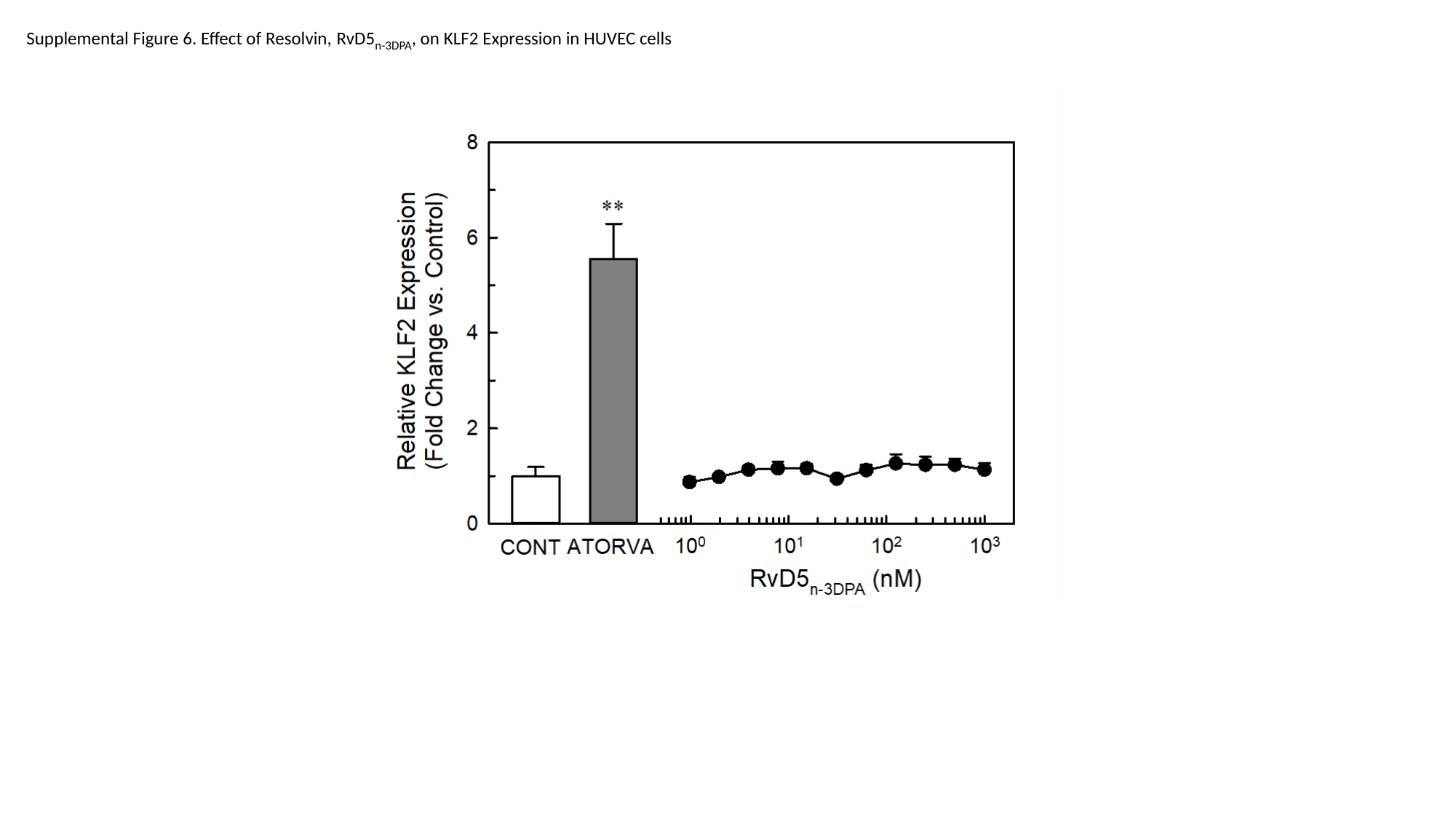

Supplemental Figure 6. Effect of Resolvin, RvD5n-3DPA, on KLF2 Expression in HUVEC cells
